# Regional fidelity vs dispersal of capelin (*Mallotus villosus*) in the Gulf of St. Lawrence, inferred from otolith chemistry

**DOI:** 10.1371/journal.pone.0352922

**Published:** 2026-09-16

**Authors:** Romaric Jac, Elisabeth Van Beveren, Olivier Le Pape, Mathieu Boudreau, Lola Coussau, Pascal Sirois, Dominique Robert, Pablo Brosset

**Affiliations:** 1 Institut des sciences de la mer, Université du Québec à Rimouski, Rimouski, Quebec, Canada; 2 UMR DECOD (Ecosystem Dynamics and Sustainability), L’Institut Agro, Ifremer, INRAE, Rennes, France; 3 Fisheries and Oceans Canada, Institut Maurice-Lamontagne, Mont-Joli, Quebec, Canada; 4 Département des Sciences Fondamentales, Université du Québec à Chicoutimi, Chicoutimi, Quebec, Canada; University of Messina, ITALY

## Abstract

Capelin (*Mallotus villosus*), a key forage fish in the Northwest Atlantic, links zooplankton to predators including commercial fishes, seabirds, and marine mammals, yet its life-cycle connectivity in the Gulf of St. Lawrence (GSL) remain poorly understood. Between 2022 and 2024, otoliths from 927 individuals collected during and after spawning were analysed by Laser Ablation Inductively Coupled Plasma Mass Spectrometry (LA-ICP-MS), and seven trace elements (Li, B, Mg, K, Zn, Sr, Ba) were used to discriminate three chemically distinct regions within the GSL. Early-life regional origin was inferred by classifying otolith core chemistry, treated as an integrated late-larval to early-juvenile signal rather than a strictly natal marker, against a discriminant model trained on otolith-edge reference signatures. Assignment of core signatures to regions indicated limited cohort-level effects, in accordance with the low interannual variability in regional otolith edge signature. A combination of widespread dispersal and patterns consistent with regional fidelity was found, with contrasts among regions in terms of fidelity vs interconnection. Fish sampled during spawning were more often reassigned to their inferred early-life region than post-spawning fish, a regional-scale homing-like pattern consistent with regional spawning fidelity. This coexistence of both dispersal and regional fidelity patterns likely contributes to a portfolio effect that may buffer the population against environmental variability and localised reproductive failures.

Forage fishes play a pivotal role in marine ecosystems worldwide, linking plankton production to higher trophic-level predators such as seabirds, marine mammals, and piscivorous fishes [1,2]. Characterised by short lifespans, rapid growth, and strong dependence on secondary production, these species often undergo pronounced “*boom-and-bust*” dynamics, with periods of high abundance followed by abrupt declines [3,4]. These natural fluctuations linked to environmental variability can be amplified by anthropogenic pressures, occasionally leading to stock collapses or long-term reductions in productivity [5–7]. Because variability in the spatial and temporal distribution of individuals plays an important role in determining how such pressures act on population trajectories, understanding habitat use and movement ecology across life stages is essential to reduce uncertainty in stock assessment, inform ecosystem-based management strategies, and anticipate climate-driven change [5,8,9]. Yet, despite recent advances in monitoring and analytical methods, substantial gaps remain in our capacity to reveal the movements, migrations, and life-history connectivity of forage species [8,10,11].

Among forage fish species, capelin (*Mallotus villosus* O.F. Müller, 1776) is one of the most important in Canadian waters [12], serving as the main prey for several commercially important fishes, seabirds, and marine mammals (e.g.[13–15],). In Canadian waters, it occurs primarily off Newfoundland and Labrador and throughout the Gulf of St. Lawrence (GSL), with smaller populations in the Canadian Arctic and in the Pacific Ocean off British Columbia [16–18]. Regional spawning-site fidelity has been hypothesised [19] alongside extensive seasonal migrations between offshore overwintering areas and inshore spawning habitats (e.g.[20,21],). Despite the weak genetic structuring reported across the North Atlantic [19,22], capelin in the GSL and on the Newfoundland-Labrador Shelf are managed as separate stocks, with the GSL itself treated as a single management unit [23].

Compared to the east Newfoundland waters, where substantial research effort has targeted capelin (e.g.[24–29],), dedicated scientific surveys and region-specific studies remain lacking in the GSL. In this area, the spawning season is spatially structured, beginning in the upper St. Lawrence estuary and progressing eastward and northward through July and August [30]. However, an accurate estimation of spawning-stock biomass continues to be hindered by limited knowledge of regional connectivity and spatial structuring, constraining robust stock assessment and management [11,23].

Insights into stock structure and movements can be gained from a wide range of approaches, including tagging, stable isotope and trace-element analyses, genetic markers, and environmental DNA [11,31,32]. Among these, otolith chemistry has proven particularly effective for tracing capelin spatial origin and reconstructing their movements [24,25,28,33,34]. Otoliths are metabolically inert, incrementally growing structures into which trace elements from surrounding waters are incorporated, creating a chronological archive of environmental exposure [35,36]. Conditions experienced during early life are recorded in the otolith core, whereas the environment at capture is reflected in the edge, allowing inference of natal origin and population connectivity [35,37,38]. This approach, however, is only as reliable as the elemental signatures it relies on are spatially distinct and temporally stable. Three hydrographically distinct regions within the GSL, the Estuary, the South, and the Strait of Belle Isle (SBI), have been shown to display such multi-year stability in their chemical fingerprints using otolith-edge chemistry [39], providing the regional framework this study builds on.

Building on this framework, the present study pursued three objectives: (i) the characterisation of the early-life regional chemical signature recorded in the otolith core, and assignment of individuals to one of these regions by classifying core signatures against a discriminant model trained on otolith-edge signatures; (ii) the comparison of inferred early-life regions with capture regions to describe regional connectivity; and (iii) the investigation of the seasonal and biological factors associated with the degree of correspondence between early-life and capture regions.

## Materials and methods

### Ethics statement

No live animals were handled or euthanised specifically for this study. All otoliths analysed were obtained from fish collected during routine DFO monitoring surveys or from commercial fishing operations conducted in accordance with applicable national and institutional regulations. Consequently, no additional animal care or ethics approval was required.

### Study area and capelin collection

**Fig 1:**
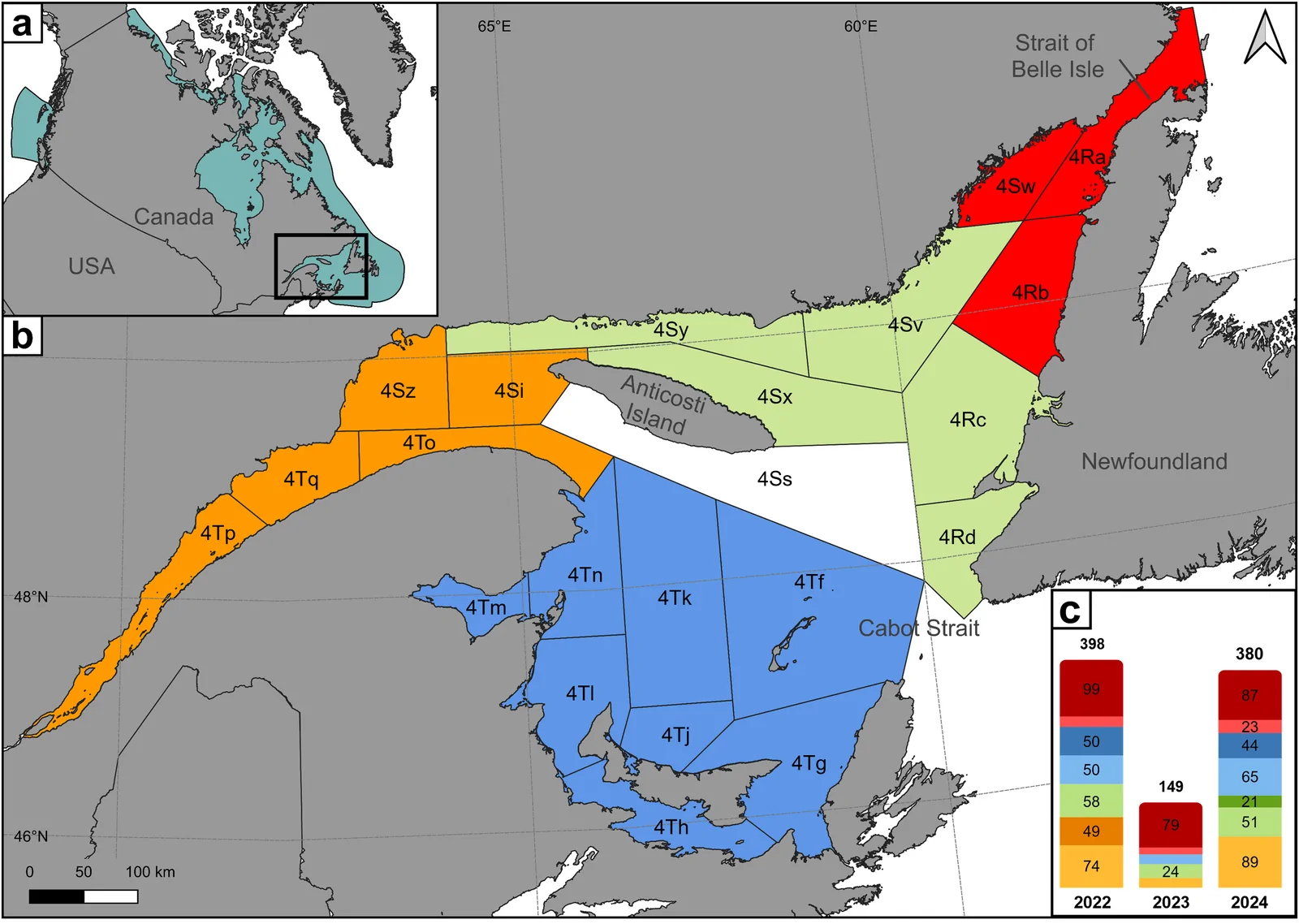
Distribution and sampling of capelin across the study area. (a) Schematic distribution of capelin across Canada’s three oceans; (b) study area with the NAFO sub-divisions and subdivided into four regions after the clustering analysis; (c) tacked bar chart displaying the number of individuals sampled per year and region. Colours represent regions as follows: orange for the Estuary, green for the North, blue for the South, and red for the Strait of Belle Isle. In the stacked bar chart, light shades represent samples from DFO multi-species bottom trawl surveys, while dark shades correspond to commercial fishery samples. Sample sizes are labelled when exceeding 20 individuals. Administrative boundaries and coastlines were obtained from geoBoundaries ([60]; https://www.geoboundaries.org), distributed under a Creative Commons Attribution 4.0 (CC BY 4.0) license. No proprietary basemap or satellite imagery was used. Map projections: (a) ESRI:102001, Canada Albers Equal Area Conic; (b) EPSG:32198, NAD83 / Québec Lambert.

The study was conducted in the St. Lawrence Estuary and Gulf (hereafter GSL; Fig 1), a semi-enclosed system with a shallow (~80 m) southern plateau and a deeper (mean ~240 m, max ~ 500 m) northern basin [40–42]. In summer, the water column is thermally stratified into a warm surface layer, a cold intermediate layer (CIL; 50–120 m) representing a key thermal habitat for capelin [43], and a deeper, warmer Atlantic-origin layer [40].

From 2022 to 2024, 927 capelin – 398 in 2022, 149 in 2023, and 380 in 2024 – were collected across the GSL using a stratified random design that balanced sampling period (during/after spawning), Northwest Atlantic Fisheries Organization (NAFO) subdivision (Fig 1), fish length, and sex. Fork length (FL) was measured, and sex was determined by external examination or gonad inspection when necessary. The dataset comprised 505 females, 356 males, and 66 individuals of undetermined sex, ranging from 7.5 to 19.0 cm FL (S1 Table).

Fish originated from two sources: commercial fisheries and scientific surveys (Fig 1). Commercial samples were collected between May and July through Fisheries and Oceans Canada (DFO) port sampling, coinciding with the spawning period [23]. Samples were obtained from nearshore fisheries using pound nets, traps, and purse seines, with spatial resolution limited to NAFO subdivisions, primarily 4Ra, 4Sw, and 4Tn (Fig 1). Scientific samples were obtained from DFO’s annual multispecies bottom trawl surveys conducted in August-September, corresponding to the post-spawning period [43], using a standardised otter trawl with precise spatial coverage across the GSL. Although sampling methods differed between sources, these differences do not affect the study objectives, as analyses focus exclusively on otolith elemental chemistry, which reflects environmental exposure rather than capture conditions [39].

### LA-ICP-MS otolith chemistry and data processing

**Fig 2:**
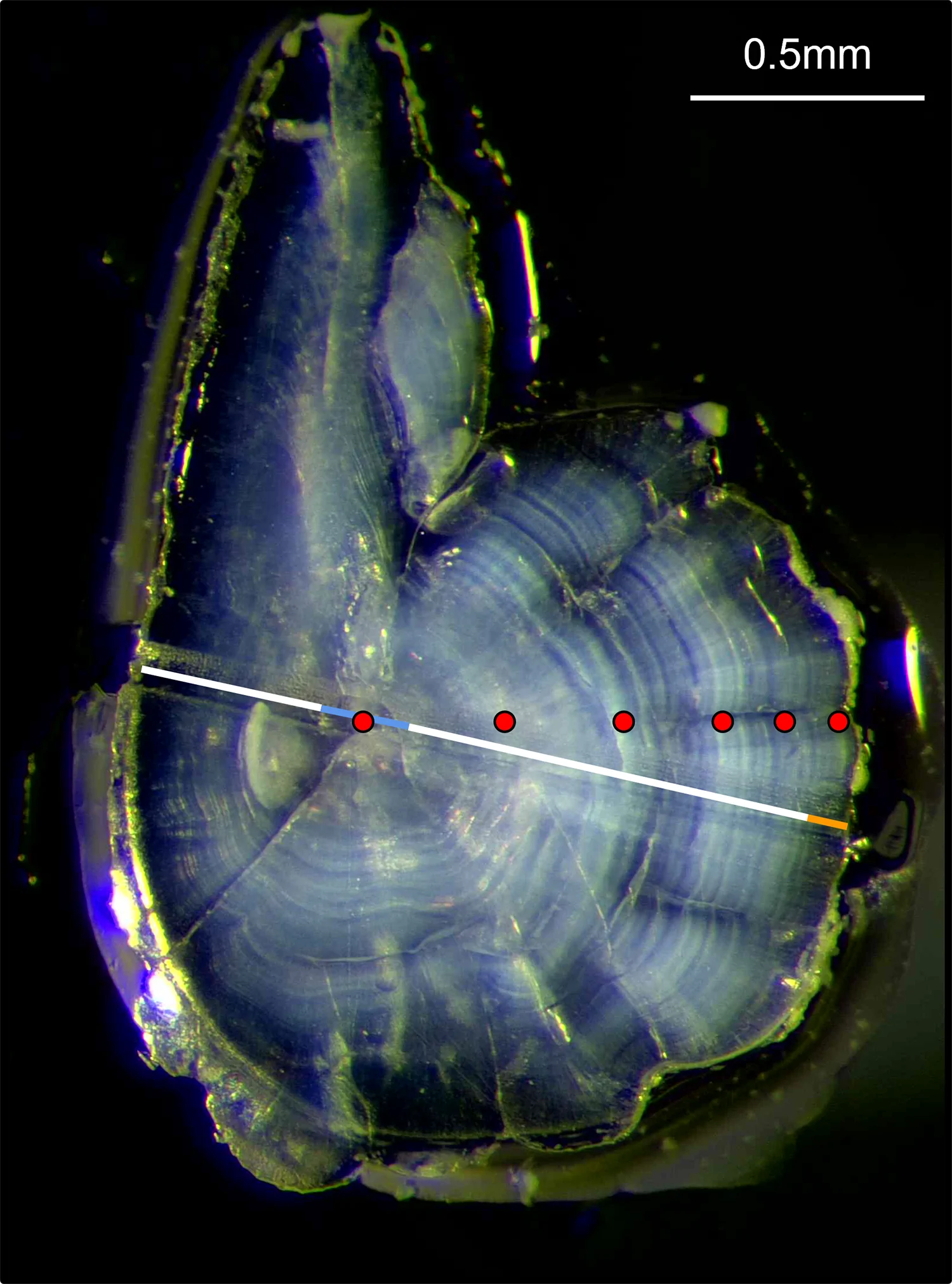
Sanded capelin otolith submerged in water following LA-ICP-MS analysis. The long white line represents the ablation transect performed during the procedure, with the white segment indicating the portion extending from the otolith core to the outer margin. The two short segments mark the core (blue) and the 40μm edge region (orange) analysed in this study. Points denote the centres of winter rings used to determine the individual’s age. Original photograph taken by the authors under a stereomicroscope and processed using ImageJ [52].

Detailed otolith preparation and LA-ICP-MS procedures are described in [39]. Briefly, sagittal otoliths were extracted following standard protocols [35], and only the right otolith of each individual was analysed. Otoliths were rinsed, cleaned, air-dried, and mounted on microscope slides using thermoplastic glue. They were ground and polished using successive abrasive papers (1200 µm, 5 µm, and 1 µm) with circular and figure-eight motions. The ventral edge of capelin otoliths is reliably exposed by this two-dimensional polishing approach, validated in previous studies [34,44]. Elemental analyses were conducted using Laser Ablation Inductively Coupled Plasma Mass Spectrometry (LA-ICP-MS; Resolution M-50 Excimer 193 nm laser coupled to an Agilent 7900x qICP-MS). For each otolith, a single continuous laser transect was ablated along the ventral growth axis, extending from the core to the outer margin (Fig 2**).** This approach captures the chronological sequence of elemental incorporation across the otolith, from early life to capture. Ablation parameters were: fluence 5 J·cm ^- 2^, repetition rate 25 Hz, beam diameter 44 µm, and scan speed 5 µm·s ^-1^. Signal stability and material removal were optimised by this beam size, while adequate temporal resolution was maintained for juvenile and adult capelin [44]. Data were acquired at 4 measurements·s^-1^. Ablated material was transported using an argon-helium gas mixture with added nitrogen, and each transect was preceded by a 30-s gas blank. A total of 38 isotopes were measured. Certified reference materials were analysed at the beginning and end of each session and after every seven otoliths to ensure analytical accuracy and precision. Measured element-to-calcium ratios were within acceptable limits relative to published reference values (USGS; [45]). Data reduction was performed using Iolite [46] in Igor Pro. Calcium (^44^Ca; 38.02% of otolith mass; Campana, 1999) as an internal standard. Only otoliths exhibiting stable calcium signals were retained. Element concentrations were expressed in parts per million (ppm), and limits of detection (LOD) were calculated as three times the standard deviation of the gas blank divided by analyte sensitivity [44].

Seven elements were retained based on their high discriminatory power: lithium (Li), boron (B), magnesium (Mg), potassium (K), zinc (Zn), strontium (Sr), and barium (Ba). Values below the LOD were replaced with LOD/2, and outliers exceeding ±5 standard deviations were replaced by the mean of adjacent measurements. Elemental concentrations were converted to element:Ca ratios, and non-normally distributed elements (Li, B, Mg, Zn) were log(x + 1)-transformed prior to statistical analyses.

The selection of these elements was detailed in [39] and included validation that spatial patterns were consistent across years. Demonstrating spatial persistence through time is a prerequisite for subsequent analyses, especially given that temporal variability can be considerable and for some elements may exceed variability in ambient water chemistry [47]. This is in part because both non-essential and essential elements were included. Essential elements (e.g., Mg and K) may be influenced by physiological regulation rather than solely reflecting ambient environmental conditions. Because inter-annual variation in these physiological processes can be substantial, the temporal stability of these elements as regional discriminators across years may be reduced. However, they can still contribute to regional discrimination if such processes exhibit persistent spatial structure. This multi-element approach is consistent with previous otolith chemistry studies on capelin, which have similarly incorporated physiologically regulated elements such as Mg, K, and Zn in multivariate analyses (e.g.[28,34],). Accordingly, the selected element suite should be interpreted as markers of regional origin rather than strictly as proxies of environmental conditions.

Two specific portions of each transect were extracted to characterise life-stage specific environmental signatures. (i) The core was identified visually and measurements within 20 µm of the otolith core (Fig 2) were used to define an early-life **chemical signature**, following established approaches for capelin and other forage fishes (e.g.[34,48,49],). Precise targeting of the core remains challenging given the convex otolith morphology, and the beam diameter inevitably integrates surrounding early post-hatch material [33,50]. Importantly, the elevated trace-element concentrations that characterise the pre-hatch and post-hatch regions of larval capelin otoliths in the GSL [49] were not recovered in the core region of the adult otoliths analysed here. The core is therefore not treated as a strictly natal (hatching) signal but rather as an integrated early-life signature, most plausibly reflecting a late-larval to early-juvenile period. (ii) The **capture signature** was defined using the outermost 40 µm of the otolith (Fig 2), which reflects the environment experienced immediately prior to capture [39,50,51].

### Age readings and age assignment

**Fig 3:**
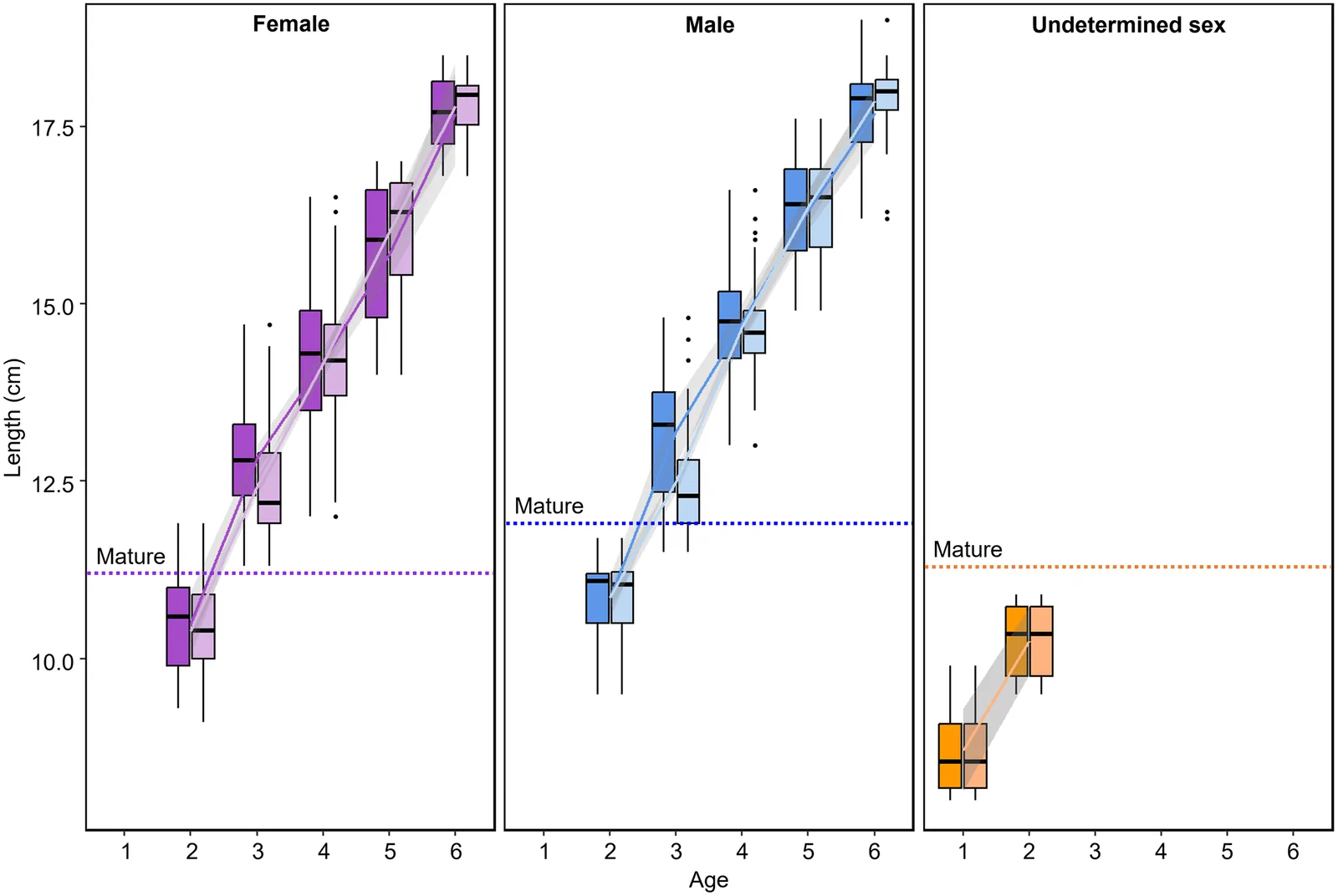
Age-Length relationship for females (pink), males (blue), and individuals of undetermined sex (orange) as well as the age–length profiles 95% confidence intervals. Dark colours indicate individuals aged directly from otoliths (Dataset_age, n = 302), while light colours represent individuals whose ages were estimated using a probabilistic Age–Length Key (pALK) based on multinomial regression (Dataset_age_supplemented, n = 594).

Capelin age was estimated by counting growth annuli on the same sagittal otoliths used for LA-ICP-MS elemental analysis. Otoliths were photographed using a Leica M125C microscope, and growth increments were measured in ImageJ ([52]; Fig 2). Unlike other regions (e.g.[33,51],), interpreting annuli from GSL capelin was challenging due to otolith opacity and the inconsistent presence of the metamorphic check. Consequently, only 307 out of 927 otoliths with clearly visible and interpretable annuli were retained for direct age determination. Age readings were independently performed by two trained readers, with disagreements resolved jointly. Otoliths for which age consensus could not be reached were excluded (n = 5; 1.63%), yielding a final age dataset of 302 individuals (*dataset_age*; Table 1). Ages ranged from 1 to 6 years (Fig 3; S1 Fig), and individuals were distributed throughout the entire study area (S2 Fig).

**Table 1:** Number of individuals per cohort, including fish aged directly from otoliths (*Dataset_age*, n = 302) or supplemented with individuals whose ages were estimated using a probabilistic Age–Length Key (pALK) (*Dataset_age_supplemented*, n = 594).

	2016	2017	2018	2019	2020	2021	2022	2023	Total
***Dataset_age***	6	23	59	42	79	71	16	6	**302**
***Dataset_age_supplemented***	9	34	128	130	132	109	46	6	**594**

To assign ages to unaged individuals, a probabilistic Age-Length Key (ALK) based on multinomial regression [53,54] was constructed using the 302 directly aged fish. Probabilities of assignment to each age class were estimated using the *multinom* function from the *nnet* package [55], generating sex-specific ALKs to account for differences in growth patterns. Unaged individuals were assigned to an age class when the posterior probability exceeded 75%. An 85.5% success rate in the reassignment of aged individuals was achieved with this threshold, supporting the ALK as a robust tool for assigning ages in population-level analyses. Based on the ageing of an additional 292 fish, the dataset was expanded to 594 individuals (*dataset_age_supplemented*; Table 1; S1 Fig), distributed throughout the entire study area (S2 Fig). Strong agreement in age-length relationships was found between the observed and ALK-augmented datasets (Fig 3), with the age distributions of newly assigned individuals closely matching those of directly aged fish, showing only minor deviations around age 3.

### Determining early-life region from otolith core chemistry

Early-life region was inferred for each individual by classifying otolith core chemical signatures, defined as the innermost 20 μm of the transect and representing an integrated early-life environmental signal [34,48,49], against a discriminant model trained on otolith-edge reference signatures, which represent the most recently deposited material before capture. This approach follows the general reassignment framework used in prior otolith-chemistry studies of stock and origin discrimination [56,57]. **Classification relied on a single multi-class** quadratic discriminant analysis (QDA**),** consistent with [39], who used this approach to establish the three-region spatial structure (Estuary, South, and SBI) within the GSL from otolith edge chemical signatures. Applying the same framework to core signatures keeps the reassignment consistent with this validated regional baseline.

Posterior probabilities of membership in each of the three regions were estimated for every core signature by the fitted QDA model, and individuals were assigned to the region with the highest probability. Assignments were retained when the maximum posterior probability exceeded 50%; a threshold above the random expectation of ~33% for three regions, ensuring that retained assignments were supported by more than chance alone. Individuals below this threshold were classified as belonging to an ‘undetermined’ region. More conservative thresholds (70% and 90%) were also evaluated; these increased the proportion of undetermined individuals but did not change the overall spatial patterns of assignment (S3 Fig), indicating the results are robust to threshold choice.

While some inter-annual variability in otolith-edge elemental composition was found by [39], spatial differences among the three regions consistently exceeded this temporal variation supporting the use of elemental signatures as reference classes across cohorts. Nonetheless, cohort effects, i.e., differences among year-classes [35,37,58,59] remained a potential confounder. To evaluate this, individuals from both datasets (*dataset_age* and *dataset_age_supplemented*) were grouped by cohort (Table 1) and classified two ways: (i) cohort-stratified, in which a separate QDA was fitted per cohort, and (ii) pooled, in which all individuals were classified together regardless of cohort. Agreement between approaches was quantified as the percentage of individuals assigned to the same early-life region under both approaches.

Inferred early-life regions of individuals caught during spawning and post-spawning were mapped to visualise regional connectivity within the GSL, using administrative boundaries and coastlines from the open-access geoBoundaries database [60], released under a CC BY 4.0 license.

### Drivers of movement between regions

Connectivity among regions of the GSL was further examined in relation to potential explanatory factors, including life stage and seasonal timing. Individuals were classified according to whether their inferred early-life and capture regions matched or differed. The probability of a regional mismatch was then analysed using a binomial generalised linear model (GLM), with status (matching versus differing regions) as the binary response variable. Maturity, sex, timing of capture (spawning versus post-spawning), and inferred early-life region were included as predictor variables. Maturity status was determined from sex-specific length thresholds: females >11.2 cm and males >11.9 cm [23]. A forward stepwise model selection procedure based on the Akaike Information Criterion (AIC; [61]) was used to identify the most parsimonious GLM.

## Results

### Cohort effects on inferred early-life region assignment

First, the influence of cohort on inferred early-life region assignment was assessed. Reassignment between age-stratified and pooled QDA models was highly consistent across regions (Table 2), indicating that cohort has little influence on assignment. Indeed, for the directly measured ages (*dataset_age*), regional concordance ranged from 74.6% to 87.0%, with an overall agreement of 80.5% between the two modelling approaches. In the dataset enriched with ALK-estimated ages (*dataset_age_supplemented*), concordance was higher across all regions, ranging from 95.7% to 97.6%, yielding an overall consistency of 97.0%. This increase likely reflects the larger sample size in the enriched dataset. Collectively, these results indicate that age class exerts a negligible effect on early-life region assignment at the scale of the entire GSL. This concordance reflects the consistency of assignments across cohorts within the present dataset rather than a formal assessment of the temporal stability of core signatures within regions. Consequently, in the absence of a detectable cohort effect, a pooled QDA model without age-class stratification was applied to all eligible individuals (n = 927) for subsequent analyses.

**Table 2:** Concordance of predicted regions by collection region, based on otolith core chemistry using quadratic discriminant analysis (QDA). Comparisons were made using both an age-class–stratified model and a pooled model to evaluate the effect of cohort on assignment outcomes. Analyses were conducted on two datasets: individuals aged directly from otoliths (*Dataset_age*, n = 302) and individuals aged via a probabilistic Age–Length Key (*Dataset_age_supplemented*, n = 594).

	Collection regions (%)				Global Consistency (%)
	Estuary	South	North	SBI	
***Dataset_age***	74.6	76.6	78.0	87.0	**80.5**
***Dataset_age_supplemented***	96.9	97.1	95.7	97.6	**97.0**

### Regional patterns of early-life region assignment

Clear differences in inferred early-life regions among capture regions were revealed by reassignment of otolith core chemistry to one of the three chemical regions, or to ‘undetermined’ when the 50% posterior probability threshold was not met (Table 3). The SBI was the most frequently assigned region. Individuals captured in the Estuary were most frequently assigned to the SBI region (38.0%), followed by the South region (24.9%) and the Estuary itself (15.7%). Capelin captured in the South were primarily assigned to their own region (31.0%) and to SBI (26.1%), whereas individuals captured in SBI were predominantly reassigned to their own region (70.4%), highlighting stronger correspondence between early-life and capture regions in this area. The North region appeared highly connected with SBI, with just under half of its individuals assigned an SBI early-life signature. The proportion of individuals with an ‘undetermined’ early-life region varied among regions. While it was relatively low in SBI (11.1%) and North (18.2%), it was higher in the Estuary (21.4%) and South (23.4%), indicating greater uncertainty in assigning individuals captured in these regions (Table 3).

**Table 3:** Reassignment results of capelin otoliths based on edge chemistry, using quadratic discriminant analysis (QDA). Values represent the percentage of individuals classified to each region (rows) assigned to each inferred early-life region (columns). Percentages corresponding to a match between capture region and inferred early-life region are highlighted in bold.

Collection region	Reassigned region (%)			
	Estuary	South	SBI	Undetermined
Estuary	**15.7**	24.9	38.0	21.4
South	19.5	**31.0**	26.1	23.4
SBI	5.4	13.2	**70.4**	11.1
North	9.7	24.7	47.4	18.2

Patterns were broadly consistent across 2022–2024 (S4 Fig), with one exception: in 2023, a larger proportion of individuals captured in the South were assigned an SBI early-life signature compared with the other two years. Elsewhere, spatial patterns remained stable across years, suggesting that the broader spatial structure is robust.

### Drivers of regional mismatch between early-life and capture regions

**Fig 4:**
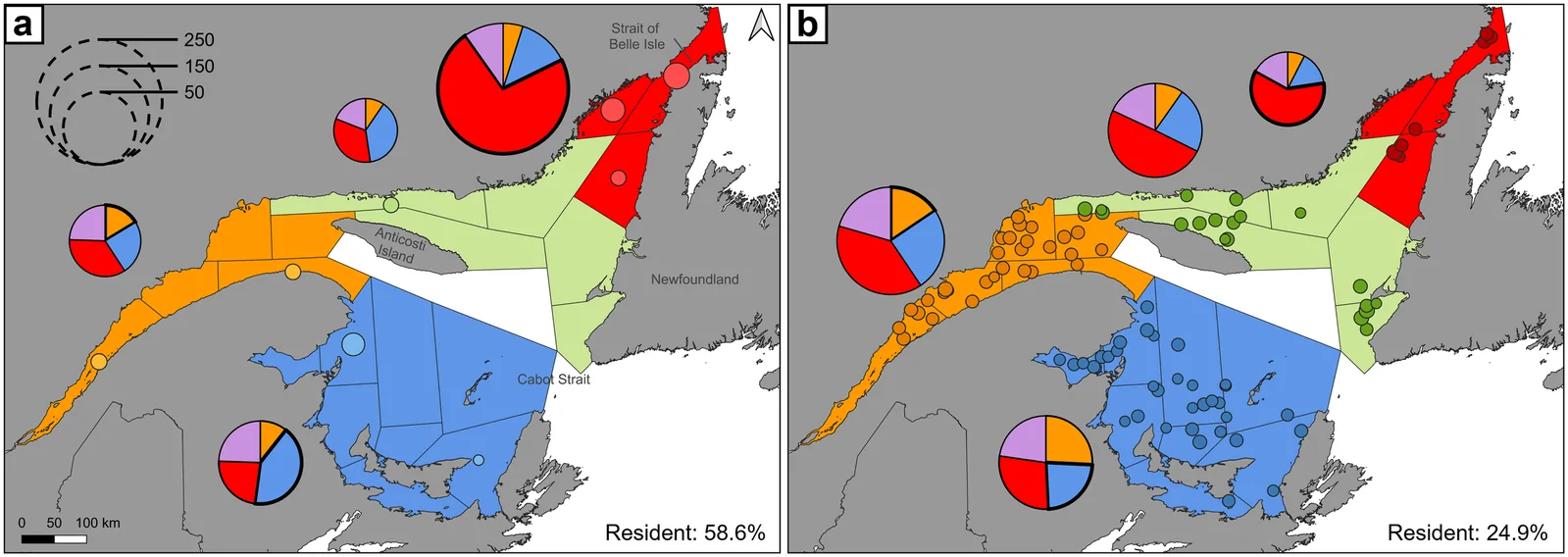
Spatial origin of capelin contingents sampled in the Gulf of St. Lawrence (a) during the spawning period and (b) after spawning. Colours indicate inferred early-life regions: orange for the Estuary, blue for the South, red for the Strait of Belle Isle and purple for an ‘undetermined’ region. Points, coloured by capture region, represent capture locations and are proportional to the number of fish captured. In the pie charts, segments with thicker outlines represent individuals whose capture region matched their inferred early-life region, whereas those with thinner outlines represent individuals whose capture and early-life regions differed. The size of each pie chart is proportional to the number of individuals captured in the area, as illustrated by the reference legend in the top left. Administrative boundaries and coastlines were obtained from geoBoundaries ([60]; https://www.geoboundaries.org), distributed under a Creative Commons Attribution 4.0 (CC BY 4.0) license. No proprietary basemap or satellite imagery was used. Map projections: EPSG:32198, NAD83 / Québec Lambert.

The probability of a regional mismatch was influenced both by survey timing and inferred early-life region (Table 4). Capelin were less likely to show a mismatch between early-life and capture regions during the spawning period than during the post-spawning period. During spawning, 58.6% of individuals were captured in regions matching their inferred early-life region, whereas only 24.9% of post-spawning individuals were captured in locations matching their inferred early-life region (Fig 4). Moreover, a lower probability of regional mismatch was found among individuals with SBI early-life signatures than among those from other regions. No significant effects of sex or maturity were detected. Overall, these results indicate that inferred early-life region and seasonal timing are associated with the degree of correspondence between early-life and capture regions.

**Table 4:** Generalised linear model (GLM) of the probability of a mismatch between inferred early-life region and capture region in capelin, as a function of maturity, spawning period (during or after), sex, and inferred early-life region determined from otolith core analysis. Model selection was based on AIC. Coefficient estimates, standard errors, z-values, and p-values are presented. Significance levels are indicated as: *** p < 0.001, ** p < 0.01, * p < 0.05.

Model Selection					
Significant predictors					AIC
NULL					992.32
Spawning period					732.67
Spawning period + Early-life origin					714.40
**Parametric coefficients**					
**Variables tested**		**Estimate**	**Std error**	**z value**	***p***
**Early-life origin**	South	−0.1035	0.3060	−0.338	
	SBI	−0.9360	0.2777	−3.370	***
**Maturity**		−0.4856	0.3342	−1.453	
**Sex**		0.2360	0.1914	1.233	
**Spawning period**		1.5612	0.1857	8.409	***

## Discussion

The present study provides the first regional-scale assessment of early-life connectivity in capelin within the GSL. Two main patterns emerged. First, capelin captured in the SBI showed the strongest correspondence between early-life and capture regions, a pattern consistent with regional spawning-site fidelity that was more pronounced during spawning than after spawning. Second, the SBI received comparatively few individuals with early-life signatures from elsewhere, whereas the Estuary and South regions showed a higher influx of individuals carrying early-life signatures from other parts of the GSL. Throughout, the otolith core is treated as an integrated early-life regional signature, reflecting the late-larval to early-juvenile period rather than a precise natal origin, so that an individual's ‘early-life region’ denotes where it resided during early life rather than where it was spawned. Given the broad, regional scale of this analysis, these patterns are unlikely to be biased by fine-scale spatial or temporal variability. Overall, these results suggest that capelin connectivity in the GSL is characterised by a combination of widespread dispersal and patterns consistent with regional fidelity, particularly in the SBI. Such dynamics create a system in which some regions are well mixed while others seem predominantly self-sustained.

### Patterns of capelin connectivity in the Gulf of St. Lawrence

Capelin in the GSL exhibit diverse and complex spatial dynamics, reflecting varying levels of regional connectivity. Overall, the population appears relatively well mixed, although mixing is less pronounced in the SBI than in other regions. At this level of connectivity, genetic differentiation is limited by extensive gene flow, consistent with earlier studies reporting broad genetic homogeneity across the region, albeit with some alleles occurring more frequently in specific areas [22,62]. During spawning, more than half of GSL capelin captured carried an early-life signature matching their capture region, consistent with regional spawning fidelity, which has previously been hypothesised for capelin [19]. The elevated correspondence during the spawning season further supports this interpretation, although the data cannot establish a return to a precise natal site, nor rule out movement away from and back to the region between early life and capture.

A notable proportion of individuals could not be confidently assigned to any of the three defined regions. This uncertainty may reflect individuals with early-life signatures from the northern zone, which lacks a sufficiently distinct elemental signature to allow high-confidence reassignments [39]. Indeed, as this zone exhibits an intermediate chemical signature between the Estuary and the SBI, some northern individuals may have been assigned to one of these two regions based on the similarity of their otolith fingerprints. Alternatively, the presence of unassigned individuals may indicate contribution from additional, unsampled regions, outside the GSL, such as the Newfoundland or the Scotian shelves [63]. Evidence for recurrent mixing between these stocks is provided by the occurrence of shared mitochondrial haplotypes across the GSL and the Newfoundland-Labrador Shelf [19,22,62]. Such exchanges are consistent with known oceanographic corridors. The SBI has been repeatedly reported as a migration route for capelin, facilitating exchanges between the GSL and the Newfoundland-Labrador Shelf [19,22], whereas the Cabot Strait serves as a corridor for pelagic fishes, including herring, mackerel, and cod [64,65].

Both the substantial proportion of capelin showing evidence of dispersal between regions and of non-assigned individuals suggest potential connectivity among regions, and outside the GSL. These connectivity patterns align with the dispersive behaviour capelin exhibit, including large-scale spawning migrations, seasonal offshore-inshore movements, and extensive feeding excursions [11,66,67]. Such dynamics facilitate colonisation of new habitats, rescue effects, and metapopulation processes, while promoting gene flow and maintaining adaptive potential under changing environmental conditions [68,69]. The findings presented here extend beyond previous genetic studies and link observed elemental signatures with the ecological processes that sustain broad-scale connectivity in and outside of the GSL through the capelin’s life cycle.

### Spawning strategies: Beach versus demersal

In the GSL, capelin exhibit two main spawning strategies: beach spawning, during which adults roll onto the shoreline, and deeper demersal spawning [17,23]. Beach spawning is better documented as it is easier to observe, and citizens can report beach-spawning events on GSL beaches [23]. In Newfoundland, these observations inform annual capelin stock assessments [26,70,71]. However, focusing solely on beach-spawning events may overlook substantial demersal spawning [72,73], emphasising the need for complementary monitoring approaches capable of detecting both spawning modes. The relative reliance on these strategies varies with local thermal conditions [74,75], with demersal spawning contributing less when spawning is delayed [75]. Previous research showed that core otolith chemistry may differ between these spawning modes [27]. In the present study, spawning sites were not directly sampled, and cores from beach and demersal spawners were not compared. Distinguishing the contributions of individual sites or spawning modes within a region is beyond the resolution of the broad-scale, edge-based framework used here. According to the methodological limits discussed above, which prevent to infer finer-scale inter-annual and among-site variation from the present analysis, our inferences are confined to the among-region scale, at which spatial structuring is pronounced [39].

During the spawning season, temperatures in the SBI, the region characterised by the coldest surface waters in the GSL, remain within the preferred range for capelin [23]. Bottom temperatures between −1 and 1 °C and the presence of a thick CIL, typically between 25 and 75 m, appear to provide extensive habitat suitable for both beach and demersal spawning [40]. Capelin captured in this region are, on average, larger and older than those from other regions (S5 Fig), consistent with patterns reported by [76]. These results indicate a strong local contribution (i.e., substantially higher than in other regions; Table 3) to spawning by fish carrying a local early-life signature and/or mature fish returning to the SBI. This pattern could reflect fitness advantages, including reducing habitat search costs, lowering predation risk, and increasing mate encounter rates [77].

Elsewhere in the GSL, surface waters are warmer, and the CIL is thinner [23,40]. In the southern GSL, bottom temperatures remain relatively cold, generally between 0 and 2 °C except in the Northumberland Strait [40]. These low temperatures, combined with the presence of shallow shelf areas, mostly shallower than 75 m, appear to provide extensive habitat suitable for demersal spawning [23]. Moreover, given the strong seasonal variability in temperature [40], capelin may favour earlier spawning at cooler subtidal sites, allowing embryos to hatch earlier and larvae to benefit from an extended growth period prior to overwintering, ultimately enhancing survival [75]. Although beach-spawning events are rarely reported in these areas [23,39], this likely reflects limited observation rather than true absence. Supporting this, systematic surveys, including mackerel egg sampling, document larval capelin in Chaleur Bay and St. George’s Bay [23], and length-frequency data indicate abundant small capelin consistent with local nursery production [76]. These findings suggest that the southern GSL provides important spawning and nursery habitats, generating a strong early-life signal despite the scarcity of reported beach-spawning events.

In the Estuary, where most previous studies within the GSL have been conducted (e.g., [78–80]), a major nursery area has been identified for capelin [81]. The Estuary is relatively warm during the spawning season (>5°C; [40]), which limits the potential for demersal spawning while still allowing beach spawning to occur [11,23,74]. Although the Estuary is of high ecological importance for capelin during the late-juvenile stage, results indicate that other regions of the GSL also play essential roles at earlier life stages.

### Larval drift and juvenile/adult migrations

Connectivity in GSL capelin may involve both larval drift and the seasonal migrations of juveniles and adults. During their relatively short larval phase, typically 1.5–3 months, depending on temperature [78,79], capelin larvae largely occupy the upper water column, where their distribution is strongly influenced by prevailing currents [82,83]. Retention has been documented in localised areas such as the Estuary [81] and coastal bays [84,85], indicating that circulation can concentrate larvae regionally. However, strong currents can transport larvae from the Estuary into the southern GSL [40,41]. The Labrador Current, entering through the SBI, also likely facilitates dispersal across the broader GSL system [86]. Despite these oceanic processes, the short larval duration relative to the spatial scale of the GSL makes larval drift unlikely to be the main driver of large-scale connectivity, although it might contribute to local recruitment variability and retention.

Beyond larval dispersal, juvenile and adult capelin undertake pronounced seasonal migrations, reflected in varying proportions of individuals showing a regional mismatch between spawning and post-spawning periods. The Estuary appears to receive substantial inflow, as many individuals sampled there carry early-life signatures from elsewhere in the GSL. Such movements require active migration and are consistent with patterns observed in the Saguenay Fjord, where individuals migrated up to 300 km during their first year [34]. Through these migrations, capelin can track favourable conditions in terms of prey availability, thermal environments, and predation pressure (e.g.[67,87,88],). From offshore wintering areas, capelin migrate inshore to spawn around July, covering distances of up to 350 km [21,89]. Males typically arrive first and remain throughout the spawning period, whereas females release eggs and then return to deeper waters [74]. Foraging migrations are closely linked to prey distribution, including copepods, amphipods, and euphausiids [90,91], and are also influenced by environmental factors such as sea temperature and ice cover [92,93]. In the SBI, the mixing of the Labrador Current and GSL outflow creates favourable trophic and thermal conditions, enhancing prey availability and extending feeding seasons [94,95], which may explain the higher proportion of individuals with matching early-life and capture regions in this region. Overwintering strategies are similarly shaped by circulation and thermal regimes. In the GSL, the CIL and the strong seasonal variability of the southern GSL restrict habitat suitability [40,43], guiding seasonal movements toward deeper thermal refuges in the Cabot Strait, the Estuary, and the SBI, consistent with overwintering patterns observed off the northeast coast of Newfoundland [96–98].

### Integrating connectivity and behaviour into capelin management

While capelin stocks in areas such as the Barents Sea, the Newfoundland-Labrador Shelf, and in Iceland are managed with explicit recognition of their ecological role [99,100], such integration through the Ecosystem Approach to Fisheries (e.g.[101,102],) is currently lacking in the GSL. The findings of this study have multiple implications for management, including clarifying the risk of localised depletion, improving interpretation of fishery and survey indicators, and guiding monitoring and survey design.

The coexistence of dispersal and patterns consistent with regional fidelity generates a portfolio effect, distributing reproductive effort across space and time and buffering populations against environmental variability, predator outbreaks, and localised reproductive failures [103,104]. Behavioural diversity has been observed in several other pelagic species (e.g., mackerel: [105]; herring: [106,107]). Maintaining this behavioural diversity is therefore essential for long-term population stability, as its erosion has been linked to collapses in small pelagic fishes [108]. Reliable estimates of early-life regional origin and population structure further improve understanding of how behavioural and spatial diversity supports the persistence of this heavily exploited stock under environmental stressors, such as altered flows or episodic disturbances [109]. However, whether this diversity confers a measurable demographic benefit, such as increased stability, resilience, or productivity, cannot be evaluated directly from the present dataset. Simulation-based population models incorporating spatial structure, connectivity, and component-specific demographic rates have proven valuable for testing portfolio-effect hypotheses in stocks with well-resolved population structure (e.g., white perch, herring; [110–112]). Applying such frameworks to capelin would first require evidence that behaviourally or spatially distinct components differ in productivity, natural mortality, growth, or maturation. This level of demographic resolution is not currently available for this stock, where genetic structuring is weak and mixing among putative components appears extensive [19,22]. Establishing this demographic differentiation is therefore a necessary precursor to any quantitative test of the portfolio-effect hypothesis in this system.

Although these patterns do not define distinct assessment units, they have clear management implications. Spatial heterogeneity can increase the risk of localised depletion, bias survey-based indices, and complicate interpretation of temporal trends [9,31]. The SBI was identified as a region with high local production and substantial commercial fishing effort, making it particularly susceptible to localised depletion and highlighting the need for spatially explicit management measures. Heterogeneous connectivity also affects the interpretation of fishery and survey indicators: temporal changes in catch-per-unit-effort (CPUE) on the west coast of Newfoundland [23] may partly reflect shifts in connectivity rather than overall stock productivity, and bottom-trawl surveys, covering only part of the GSL, cannot be interpreted independently without accounting for regional movements and mixing. Incorporating connectivity information therefore reduces the risk of biased stock assessments due to localised signals [113].

Survey design and monitoring programmes should aim for broad spatial coverage to capture the full spectrum of population composition, life stages, and regional contributions, ensuring that derived indicators reflect stock-wide dynamics rather than localised subunits. In this context, this analysis provides empirical evidence of substantial connectivity across the GSL, a feature previously suspected but undocumented. By linking individual movement histories and inferred early-life regions to spatial population structure, these findings enhance understanding of capelin stock dynamics, inform ecosystem-based management, and support the development of more robust survey designs and future research directions.

### Uncertainty and limitations

Otolith elemental composition reflects a complex interplay between environmental conditions (e.g., temperature, salinity, productivity) and intrinsic physiological processes (e.g., growth rate, metabolism), and both must be accounted for when reconstructing environmental histories [47,114–116]. A central question for the present study is which life-history window the otolith core records. In capelin, larval otoliths display markedly elevated trace-element concentrations in both the pre-hatch and post-hatch regions, corresponding to the first months of life [28,33,49,51], yet these elevated concentrations were not recovered in the capelin otoliths cores analysed here. The most parsimonious interpretation is that the core does not capture the larval period as strictly defined, but rather an integrated window spanning the late-larval to early-juvenile (settlement) phase, the underlying mechanisms remaining unresolved [49]. Importantly, this uncertainty about the precise period recorded does not diminish the value of the core signal: even without identifying where individuals were spawned, the core integrates a formative early-life window, encompassing the growth and survival processes that shape recruitment and future stock dynamics, and therefore remains ecologically meaningful as an integrated early-life regional signature rather than a strict natal marker.

A further consideration is temporal stability. Multi-year stability of region-specific signatures has been demonstrated on the otolith edge, which reflects the adult capture environment [39,117], but the stability of core signatures within regions was not directly tested here. This distinction matters because the early-life window is laid down by larvae and early juveniles, whose physiology and habitat differ from those of the adults on which the regional baseline was validated, so edge stability cannot be assumed to carry over directly to the core. At finer scales, inter-annual variation in larval otolith chemistry and substantial among-site heterogeneity has both been documented for capelin and can reduce discrimination success [28,49]. Together, these constraints confine our inferences to the broad, region-level scale and preclude individual-level natal assignment. Despite these limitations, several lines of evidence indicate that the broad-scale patterns reported here are robust. First, the assignments are anchored to otolith-edge signatures whose multi-year stability and strong spatial contrast are established, with among-region differences consistently exceeding inter-annual variation [39]. attributed these patterns primarily to large-scale, temporally persistent drivers rather than to ephemeral local conditions. Because such drivers are stable over time, the among-region contrast they generate was likely expressed during the early-life period as well, lending indirect support to the use of the core signal for broad regional assignment. Second, the high concordance between age-stratified and pooled models (80.5% for directly aged fish; 97.0% for the age-supplemented dataset) indicates that cohort effects have limited influence on assignments, reflecting internal consistency across cohorts. Third, the consistently low proportion of ‘undetermined’ individuals is informative: because the three regions are strongly chemically differentiated [39], a core signature incompatible with all three falls below the assignment threshold and is classified as ‘undetermined’ rather than forced into an incorrect region, so the low frequency of such individuals indicates that most core signatures are compatible with a single regional signature.

As a next step, an exploratory clustering analysis of early-life otolith signatures could help determine whether individuals of undetermined origin form a distinct group, or an intermediate group potentially associated with the northern zone. Additionally, the development of a more comprehensive otolith biobank, incorporating reference signatures from adjacent and currently unsampled regions, could help resolve these assignment ambiguities in future work [11,39].

Finally, the applicability of this framework is system-dependent. The environmental stability of the GSL during summer relative to the species’ short lifespan, together with the broad spatial scale of the analysis relative to the scale of physiological and environmental variability, supports the use of this core-classification approach to detect large-scale connectivity. Caution is warranted, however, when extending the approach to finer spatial scales, to more dynamic environments, or to longer-lived species.

## Supporting information

S1 Table Summary of capelin sampling in the Gulf of St. Lawrence (2022–2024), organised by captured region, year, and spawning phase. For each stratum, sex composition and the distributions of fork length and age are reported; age values were calculated after reattribution using the Age-Length Key.
(DOCX)

S1 Fig Distribution of capelin ages, determined (a) by direct observation and (b) using a probabilistic Age–Length Key (ALK), shown for the four capture areas across the three sampling years. (DOCX)

S2 Fig Spatial distribution of aged capelin determined (a) through direct observation and (b) using a probabilistic Age–Length Key (ALK). Circle size indicates the number of capelin sampled per station. Administrative boundaries and coastlines were obtained from geoBoundaries ([60]; https://www.geoboundaries.org), distributed under a Creative Commons Attribution 4.0 (CC BY 4.0) license. No proprietary basemap or satellite imagery was used. Map projections: EPSG:32198, NAD83 / Québec Lambert.
(DOCX)

S3 Fig Spatial origin of capelin contingents sampled in the Gulf of St. Lawrence inferred using three probability thresholds. For each individual, the region with the highest assignment probability was selected: (a) > 50%, (b) > 70%, and (c) > 90%. Colours indicate inferred early-life regions: orange for the Estuary, blue for the South, red for the Strait of Belle Isle and purple for an ‘undetermined’ region. Points, coloured by capture region, represent capture locations and are proportional to the number of fish captured. In the pie charts, segments with thicker outlines represent individuals whose capture region matched their inferred early-life region, whereas those with thinner outlines represent individuals whose capture and early-life regions differed. The size of each pie chart is proportional to the number of individuals captured in the area, as illustrated by the reference legend in the top left. Administrative boundaries and coastlines were obtained from geoBoundaries ([60]; https://www.geoboundaries.org), distributed under a Creative Commons Attribution 4.0 (CC BY 4.0) license. No proprietary basemap or satellite imagery was used. Map projections: EPSG:32198, NAD83 / Québec Lambert.
(DOCX)

S4 Fig Spatial origin of capelin contingents sampled in the Gulf of St. Lawrence inferred for the three sampling years: (a) 2022, (b) 2023, and (c) 2024. Colours indicate inferred early-life regions: orange for the Estuary, blue for the South, red for the Strait of Belle Isle and purple for an ‘undetermined’ region. Points, coloured by capture region, represent capture locations and are proportional to the number of fish captured. In the pie charts, segments with thicker outlines represent individuals whose capture region matched their inferred early-life region, whereas those with thinner outlines represent individuals whose capture and early-life regions differed. The size of each pie chart is proportional to the number of individuals captured in the area, as illustrated by the reference legend in the top left. Administrative boundaries and coastlines were obtained from geoBoundaries ([60]; https://www.geoboundaries.org), distributed under a Creative Commons Attribution 4.0 (CC BY 4.0) license. No proprietary basemap or satellite imagery was used. Map projections: EPSG:32198, NAD83 / Québec Lambert.
(DOCX)

S5 Fig Age distribution of capelin by inferred early-life region (Estuary, South, SBI, and Undetermined) across the four capture areas (Estuary, South, North, and SBI), with the overall global age distribution shown in the background. (DOCX)
