# Supplementary material for: Regional fidelity vs dispersal of capelin (Mallotus villosus) in the Gulf of St. Lawrence, inferred from otolith chemistry
Source: PLoS One. 2026 Sep 16;21(9):e0352922. doi: 10.1371/journal.pone.0352922 (PMC13580995; doi:10.1371/journal.pone.0352922)
Supplement: Supplementary file 1 [file pone.0352922.s009.pdf]

Response to reviewers

We would like to sincerely thank the reviewers for their careful evaluation and constructive feedback, which have greatly contributed to improving the quality and clarity of our manuscript. We have carefully considered all comments and made the corresponding revisions throughout the text. In our responses below, line numbers refer to the revised, clean version of the manuscript (i.e., without tracked changes).

Reviewer 1

General comments

The authors undertake an ambitious application of otolith chemistry in understanding natal homing by a key forage fish, capelin, within the large Gulf of St. Lawrence system. They navigate adroitly the issue of system/population size through tactics that link adult to early life chemical signatures. A key assumption that across annual cohorts, the early life signature is stable and regrettably could not be tested within the frame of the study. Still the authors sufficiently communicate this limitation throughout the paper. The paper is well presented and analysis appropriate to the study objectives. I found very little to improve in the paper

Answer: We thank the reviewer for their positive assessment of our study and agree that the temporal stability of the early-life (core) chemical signature is a key premise of our approach.

Specific comments

It only became clear to me late in the discussion why more robust and flexible machine learning approaches were not used in classifying individuals. Rather a more standard finite stock mixture approach was used – QDA. I think it would be helpful to present this justification earlier in Methods.

Answer: We agree that the rationale for using QDA reads more naturally where the method is first introduced rather than emerging later in the Discussion. We have added this justification to the Methods (lines 197-200).

The section on integrating connectivity (partial migration) into management (l 436) merits including but the question of how to assess whether this diversity really matters is left untreated. Here there are ways forward through simulation analyses. I list several related citations below that bear on the portfolio effect:

Kerr, L.A., Cadrin, S.X., Secor, D.H., 2010. The role of spatial dynamics in the stability, resilience, and productivity of an estuarine fish population. Ecological Applications, 20(2): 497-507.

Kerr, L.A., Cadrin, S.X., Secor, D.H., Taylor, N.G., 2017. Modeling the implications of stock mixing and life history uncertainty of Atlantic bluefin tuna. Canadian Journal of Fisheries and Aquatic Sciences, 74(11): 1990-2004.

Secor, D.H., Kerr, L.A., Cadrin, S.X., 2009. Connectivity effects on productivity, stability, and persistence in a herring metapopulation model. ICES Journal of Marine Science, 66(8): 1726-1732.

Answer: We thank the reviewer for these relevant references and agree that a quantitative test of whether behavioural/spatial diversity confers a demographic benefit would strengthen this discussion. Simulation-based population models such as those of Secor et al. (2009), Kerr et al. (2010), and Kerr et al. (2017) are powerful tools for testing portfolio-effect hypotheses, but in each of these applications the modelled components are demographically distinct, with vital rates (productivity, natural mortality, recruitment, growth, maturation) that can be estimated or reasonably assumed to differ. For capelin in the GSL, we do not currently have evidence for this level of discreteness: genetic structuring is weak (Cayuela et al., 2020; Kenchington et al., 2015), and behavioural mixing/switching among putative components appears more extensive than in the systems these models were built for. Parameterising a simulation under these conditions would require assuming differential productivity among components largely a priori, which we felt would not yield a robust test of the portfolio-effect hypothesis at this stage. We have revised the manuscript (lines 410-428) to cite these approaches while clarifying that establishing demographic differentiation among components is a necessary first step before such simulation-based methods become applicable to this stock.

Reviewer 2

General comments

I do not agree with classifying individuals as “migratory” or “resident” within the scope of this work. The analysis compares inferred early-life regional origin with capture region during or after spawning but does not describe movement throughout the individuals’ lives. Individuals whose inferred early-life and capture regions match may still have migrated before returning to spawn. Therefore, terms related to “dispersal” or “patterns consistent with regional spawning-site fidelity,” which are also used in the manuscript, may be more appropriate.

Answer: We thank the reviewer for this important point to which we agree. Our two-point comparison (inferred early-life region vs. capture region) cannot describe movement across an individual’s full life history, and a matching region does not rule out movement away from, or back to, that region. We have removed the “resident”/“migrant” classification throughout the manuscript and replaced it with language describing only what was measured: whether early-life and capture regions matched or differed. Where relevant to interpretation, we now use “dispersal” and “patterns consistent with regional spawning-site fidelity,” as suggested, rather than implying a fixed behavioural status.

In my view, the main findings of this work concern patterns consistent with regional spawning-site fidelity and the contribution of individuals with early-life signatures assigned to the SBI region (lines 306–314 and 323–325). The opening of the Discussion could focus more clearly on these findings, as the claims in lines 301–303 appear broader than the scope of the analysis.

Answer: We agree that the original opening framed the study’s scope more broadly (as a first large-scale assessment of movement patterns) than the analysis supports, and buried the two central findings: (i) patterns consistent with regional spawning-site fidelity and (ii) the distinct local contribution in the SBI. We have restructured the opening of the Discussion (lines 279-292) to foreground these two findings directly.

Given the importance of spawning-site fidelity to the study’s findings, I felt that this topic could be developed more fully in the Introduction. This could include further discussion of the importance of understanding the species’ spawning ecology, as well as the advantages and limitations of using otolith chemistry to investigate early-life regional origin and spawning-site fidelity.

Answer: We have added text situating spawning-site fidelity as a behavioural process that can persist independently of gene flow and that carries potential fitness benefits in the introduction (lines 54-58). We have also added a brief statement on the advantages of otolith chemistry for investigating early-life origin in a species in lines 65-74.

The term “edge-to-core approach,” used for instance in lines 23, 90 and 209, may lead readers to assume that the complete life history of each individual was reconstructed along the entire otolith

transect. Please revise this terminology throughout the manuscript and reduce the emphasis placed on the edge-to-core approach to represent the scope of the analysis accurately.

Answer: We agree that repeated use of "edge-to-core approach" as a named method risked suggesting reconstruction of an individual's complete life history across the full otolith transect, rather than the classification of a single core signature against an edge-trained reference model. We have revised the terminology throughout the manuscript. The method is now introduced in the Methods section, with an explicit description of what it does; subsequent mentions use the plainer term "core-classification approach" rather than repeating "edge-to-core" as a standalone label.

Specific Comments

I found the Methods section somewhat lengthy and presenting some of the information more concisely could improve readability. For example, the description of the study regions could be moved to the Introduction and presented alongside the regional otolith-chemistry patterns identified by Jac et al. (2026).

Answer: We have shortened the Methods section overall.

It would also be helpful to include a descriptive table summarizing the number of samples by region and year, including spawning phase (spawning or post-spawning), sex, and the mean, minimum and maximum fish length and age. A table similar to the information presented in S1 Fig would allow readers to understand the sampling design more easily.

Answer: We have added a descriptive table (Table S1) summarising sample sizes by region, year, and spawning phase, along with sex composition and the distributions of fork length and age.

The Methods state that this study represents the second attempt to estimate the age of capelin in the GSL (lines 186–188), but the resulting age estimates are not clearly reported or discussed. Please present these results or clarify the relevance of this statement.

Answer: As the resulting age estimates were not the focus of this study and were not discussed further, we have removed this sentence for clarity.

I think it is important to emphasize more clearly that samples were collected from four areas but assigned to only three classification regions because the chemical signature of the North could not be distinguished from those of the other regions. Although this information is present in the manuscript, a clear statement in the Methods, and consistent clarification elsewhere, would help readers follow the analysis and interpretation.

Answer: We have added an explicit statement at the start of the classification section (lines 192-200) clarifying that although samples were collected from four areas, the North could not be reliably distinguished from the other regions based on its chemical signature (Jac et al. 2026, cited in the paper) and was therefore not retained as a separate classification region, and we have carried this clarification through consistently elsewhere in the manuscript.

In the interannual comparison (S4 Fig; lines 287–288), the predominant inferred early-life region of fish captured in the South changes among years, which should be reported in the Results.

Answer: We have revised the relevant sentence (lines 262-264) to explicitly report this exception, i.e., that the predominant inferred early-life region of fish captured in the South differed from the general pattern in 2023.

Although the text explains why sex and maturity were excluded from the final model, I suggest adding their model-selection results to Table 4 so that readers can assess their contribution directly.

Answer: The model-selection results for sex and maturity have been added to Table 4.

Please include the temperature range in line 394.

Answer: The temperature range has been added.

In the supplementary maps, such as S3 Fig, please clarify that the points are coloured according to capture site.

Answer: We have clarified this point in the relevant figure captions, which now read: "Points, coloured by capture region, represent capture locations and are proportional to the number of fish captured."

Finally, the authors could consider an exploratory clustering analysis of early-life signatures to examine whether fish of undetermined origin form distinct groups or an intermediate group, potentially associated with the North.

Answer: While a full clustering analysis is beyond the scope of the present study, we agree that this is a promising direction for future work. We have added text in the "*Uncertainty and limitations*" section of the Discussion (lines 487-491).
