# Supplementary material for: Regional fidelity vs dispersal of capelin (Mallotus villosus) in the Gulf of St. Lawrence, inferred from otolith chemistry
Source: PLoS One. 2026 Sep 16;21(9):e0352922. doi: 10.1371/journal.pone.0352922 (PMC13580995; doi:10.1371/journal.pone.0352922)
Supplement: Supplementary file 2 [file pone.0352922.s001.docx]

|  | **Spawning phase** | **Females/Males/Undetermined** | **Mean FL (cm)** | **FL range (cm)** | **Mean age** | **Age range** |
| --- | --- | --- | --- | --- | --- | --- |
| **Estuary** | | | | | | |
| 2022 | Spawning | 25 / 24 | 14.3 | 12.0 - 16.7 | 4.0 | 3 - 5 |
|  | Post-spawning | 29 / 38 /7 | 12.5 | 9.4 - 15.0 | 3.1 | 2 - 4 |
| 2023 | Post-spawning | 6 / 7 / 4 | 10.9 | 8.7 - 15.5 | 2.3 | 2 - 4 |
| 2024 | Post-spawning | 71 / 12 / 6 | 12.7 | 8.0 - 15.4 | 3.2 | 1 - 5 |
| **North** | | | | | | |
| 2022 | Post-spawning | 25 / 32 / 1 | 12.6 | 10.9 - 14.9 | 3.3 | 2 - 5 |
| 2023 | Post-spawning | 13 / 6 / 5 | 11.7 | 9.9 - 15.0 | 2.3 | 2 - 3 |
| 2024 | Spawning | 8 / 13 | 14.4 | 12.3 - 16.2 | 3.9 | 3 - 5 |
|  | Post-spawning | 33 / 5 / 13 | 11.6 | 7.5 - 14.6 | 3 | 1 - 4 |
| **South** | | | | | | |
| 2022 | Spawning | 24 / 26 | 14.1 | 11.7 - 17.2 | 3.7 | 3 - 5 |
|  | Post-spawning | 25 / 12 / 15 | 11.5 | 7.5 - 15.6 | 2.9 | 2 - 5 |
| 2023 | Post-spawning | 13 / 4 | 14.8 | 12.8 - 17.0 | 4 | 3 - 5 |
| 2024 | Spawning | 9 / 33 / 2 | 14.0 | 10.5 - 16.4 | 3.7 | 3 - 5 |
|  | Post-spawning | 38 / 22 / 5 | 12.3 | 8.0 - 15.9 | 2.8 | 2 - 5 |
| **SBI** | | | | | | |
| 2022 | Spawning | 57 / 42 | 15.2 | 11.5 - 18.3 | 4.5 | 3 - 6 |
|  | Post-spawning | 10 / 7 / 1 | 12.8 | 11.8 - 13.4 | 3.4 | 3 - 4 |
| 2023 | Spawning | 46 / 32 / 1 | 15.2 | 10.7 - 18.5 | 4.3 | 2 - 6 |
|  | Post-spawning | 7 / 5 | 12.6 | 10.5 - 15.1 | 2.7 | 2 – 5 |
| 2024 | Spawning | 50 / 37 | 15.0 | 10.6 - 19.0 | 4.3 | 2 – 6 |
|  | Post-spawning | 18 / 2 / 3 | 13.7 | 7.5 - 16.8 | 3.8 | 1 - 5 |

**S1 Table. Summary of capelin sampling in the Gulf of St. Lawrence (2022–2024)**, organised by captured region, year, and spawning phase. For each stratum, sex composition and the distributions of fork length and age are reported; age values were calculated after reattribution using the Age-Length Key.
