# Supplementary material for: Regional fidelity vs dispersal of capelin (Mallotus villosus) in the Gulf of St. Lawrence, inferred from otolith chemistry
Source: PLoS One. 2026 Sep 16;21(9):e0352922. doi: 10.1371/journal.pone.0352922 (PMC13580995; doi:10.1371/journal.pone.0352922)
Supplement: Supplementary file 3 [file pone.0352922.s002.docx]

**S1 Fig. Distribution of capelin ages**, determined (a) by direct observation and (b) using a probabilistic Age–Length Key (ALK), shown for the four capture areas across the three sampling years.
