# Supplementary material for: Regional fidelity vs dispersal of capelin (Mallotus villosus) in the Gulf of St. Lawrence, inferred from otolith chemistry
Source: PLoS One. 2026 Sep 16;21(9):e0352922. doi: 10.1371/journal.pone.0352922 (PMC13580995; doi:10.1371/journal.pone.0352922)
Supplement: Supplementary file 4 [file pone.0352922.s003.docx]

**S2 Fig. Spatial distribution of aged** capelin determined (a) through direct observation and (b) using a probabilistic Age–Length Key (ALK). Circle size indicates the number of capelin sampled per station. Administrative boundaries and coastlines were obtained from geoBoundaries ([60]; <https://www.geoboundaries.org>), distributed under a Creative Commons Attribution 4.0 (CC BY 4.0) license. No proprietary basemap or satellite imagery was used. Map projections: EPSG:32198, NAD83 / Québec Lambert.
