# Supplementary material for: Regional fidelity vs dispersal of capelin (Mallotus villosus) in the Gulf of St. Lawrence, inferred from otolith chemistry
Source: PLoS One. 2026 Sep 16;21(9):e0352922. doi: 10.1371/journal.pone.0352922 (PMC13580995; doi:10.1371/journal.pone.0352922)
Supplement: Supplementary file 6 [file pone.0352922.s005.docx]

**S4 Fig. Spatial origin of capelin contingents sampled in the Gulf of St. Lawrence inferred for the three sampling years: (a) 2022, (b) 2023, and (c) 2024.** Colours indicate inferred early-life regions: orange for the Estuary, blue for the South, red for the Strait of Belle Isle and purple for an ‘undetermined’ region. Points, coloured by capture region, represent capture locations and are proportional to the number of fish captured. In the pie charts, segments with thicker outlines represent individuals whose capture region matched their inferred early-life region, whereas those with thinner outlines represent individuals whose capture and early-life regions differed. The size of each pie chart is proportional to the number of individuals captured in the area, as illustrated by the reference legend in the top left. Administrative boundaries and coastlines were obtained from geoBoundaries ([60]; <https://www.geoboundaries.org>), distributed under a Creative Commons Attribution 4.0 (CC BY 4.0) license. No proprietary basemap or satellite imagery was used. Map projections: EPSG:32198, NAD83 / Québec Lambert.
