# Supplementary material for: Regional fidelity vs dispersal of capelin (Mallotus villosus) in the Gulf of St. Lawrence, inferred from otolith chemistry
Source: PLoS One. 2026 Sep 16;21(9):e0352922. doi: 10.1371/journal.pone.0352922 (PMC13580995; doi:10.1371/journal.pone.0352922)
Supplement: Supplementary file 7 [file pone.0352922.s006.docx]

**S5 Fig. Age distribution of capelin by inferred early-life region** (Estuary, South, SBI, and Undetermined) across the four capture areas (Estuary, South, North, and SBI), with the overall global age distribution shown in the background.
