# Supplementary material for: Regional fidelity vs dispersal of capelin (Mallotus villosus) in the Gulf of St. Lawrence, inferred from otolith chemistry
Source: PLoS One. 2026 Sep 16;21(9):e0352922. doi: 10.1371/journal.pone.0352922 (PMC13580995; doi:10.1371/journal.pone.0352922)
Supplement: Supplementary file 8 [file pone.0352922.s007.docx]

**PLOS One Review: Regional connectivity and partial residency of capelin (Mallotus villosus) in the Gulf of St. Lawrence inferred from otolith chemistry**

Overall, I found the manuscript well written and the study valuable for understanding the spatial structure and spawning ecology of capelin in the Gulf of St. Lawrence. The large otolith dataset provides important insights into the relationship between inferred early-life origin and capture regions during and after spawning. My main comments concern the terminology and scope of the movement-related interpretations, as well as some aspects that could be clarified. These comments are provided below.

**General comments**

I do not agree with classifying individuals as “migratory” or “resident” within the scope of this work. The analysis compares inferred early-life regional origin with capture region during or after spawning but does not describe movement throughout the individuals’ lives. Individuals whose inferred early-life and capture regions match may still have migrated before returning to spawn. Therefore, terms related to “dispersal” or “patterns consistent with regional spawning-site fidelity,” which are also used in the manuscript, may be more appropriate.

In my view, the main findings of this work concern patterns consistent with regional spawning-site fidelity and the contribution of individuals with early-life signatures assigned to the SBI region (lines 306–314 and 323–325). The opening of the Discussion could focus more clearly on these findings, as the claims in lines 301–303 appear broader than the scope of the analysis.

Given the importance of spawning-site fidelity to the study’s findings, I felt that this topic could be developed more fully in the Introduction. This could include further discussion of the importance of understanding the species’ spawning ecology, as well as the advantages and limitations of using otolith chemistry to investigate early-life regional origin and spawning-site fidelity.

The term “edge-to-core approach,” used for instance in lines 23, 90 and 209, may lead readers to assume that the complete life history of each individual was reconstructed along the entire otolith transect. Please revise this terminology throughout the manuscript and reduce the emphasis placed on the edge-to-core approach to represent the scope of the analysis accurately.

**Specific comments**

I found the Methods section somewhat lengthy, and presenting some of the information more concisely could improve readability. For example, the description of the study regions could be moved to the Introduction and presented alongside the regional otolith-chemistry patterns identified by Jack et al. (2026).

It would also be helpful to include a descriptive table summarizing the number of samples by region and year, including spawning phase (spawning or post-spawning), sex, and the mean, minimum and maximum fish length and age. A table similar to the information presented in S1 Fig would allow readers to understand the sampling design more easily.

The Methods state that this study represents the second attempt to estimate the age of capelin in the GSL (lines 186–188), but the resulting age estimates are not clearly reported or discussed. Please present these results or clarify the relevance of this statement.

I think it is important to emphasize more clearly that samples were collected from four areas but assigned to only three classification regions because the chemical signature of the North could not be distinguished from those of the other regions. Although this information is present in the manuscript, a clear statement in the Methods, and consistent clarification elsewhere, would help readers follow the analysis and interpretation.

In the interannual comparison (S4 Fig; lines 287–288), the predominant inferred early-life region of fish captured in the South changes among years, which should be reported in the Results.

Although the text explains why sex and maturity were excluded from the final model, I suggest adding their model-selection results to Table 4 so that readers can assess their contribution directly.

Please include the temperature range in line 394.

In the supplementary maps, such as S3 Fig, please clarify that the points are coloured according to capture site.

Finally, the authors could consider an exploratory clustering analysis of early-life signatures to examine whether fish of undetermined origin form distinct groups or an intermediate group, potentially associated with the North.
