# Supplementary material for: Regional fidelity vs dispersal of capelin (Mallotus villosus) in the Gulf of St. Lawrence, inferred from otolith chemistry
Source: PLoS One. 2026 Sep 16;21(9):e0352922. doi: 10.1371/journal.pone.0352922 (PMC13580995; doi:10.1371/journal.pone.0352922)
Supplement: Peer review history

# Peer review: Decision Letter 0

**DOI:** 10.1371/journal.pone.0352922.r001

**Authors:** Claudio D'Iglio

21 Jul 2026

Dear Dr. Jac,

Thank you for submitting your manuscript to PLOS ONE. After careful consideration, we feel that it has merit but does not fully meet PLOS ONE’s publication criteria as it currently stands. Therefore, we invite you to submit a revised version of the manuscript that addresses the points raised during the review process.

Please follow the reviewers suggestions, in order to fix the minor concerns raised during the revision process.

Please submit your revised manuscript by Aug 20 2026 11:59PM. If you will need more time than this to complete your revisions, please reply to this message or contact the journal office at plosone@plos.org. When you're ready to submit your revision, log on to https://www.editorialmanager.com/pone/ and select the 'Submissions Needing Revision' folder to locate your manuscript file.

- A letter that responds to each point raised by the academic editor and reviewer(s). You should upload this letter as a separate file labeled 'Response to Reviewers'.
- A marked-up copy of your manuscript that highlights changes made to the original version. You should upload this as a separate file labeled 'Revised Manuscript with Track Changes'.
- An unmarked version of your revised paper without tracked changes. You should upload this as a separate file labeled 'Manuscript'.

If you would like to make changes to your financial disclosure, please include your updated statement in your cover letter. Guidelines for resubmitting your figure files are available below the reviewer comments at the end of this letter.

If applicable, we recommend that you deposit your laboratory protocols in protocols.io to enhance the reproducibility of your results. Protocols.io assigns your protocol its own identifier (DOI) so that it can be cited independently in the future. For instructions see: https://journals.plos.org/plosone/s/submission-guidelines#loc-laboratory-protocols. Additionally, PLOS ONE offers an option for publishing peer-reviewed Lab Protocol articles, which describe protocols hosted on protocols.io. Read more information on sharing protocols at https://plos.org/protocols?utm_medium=editorial-email&utm_source=authorletters&utm_campaign=protocols.

As the corresponding author, your ORCID iD is verified in the submission system and will appear in the published article. PLOS supports the use of ORCID, and we encourage all coauthors to register for an ORCID iD and use it as well. Please encourage your coauthors to verify their ORCID iD within the submission system before final acceptance, as unverified ORCID iDs will not appear in the published article. *Only*  the individual author can complete the verification step; PLOS staff *cannot*  verify ORCID iDs on behalf of authors.

We look forward to receiving your revised manuscript.

Kind regards,

Claudio D'Iglio, Ph.D.

Academic Editor

PLOS One

**Journal Requirements:**

When submitting your revision, we need you to address these additional requirements.

1. Please ensure that your manuscript meets PLOS ONE's style requirements, including those for file naming. The PLOS ONE style templates can be found at

https://journals.plos.org/plosone/s/file?id=wjVg/PLOSOne_formatting_sample_main_body.pdf and

https://journals.plos.org/plosone/s/file?id=ba62/PLOSOne_formatting_sample_title_authors_affiliations.pdf

2. In your Methods section, please provide additional information regarding the permits you obtained for the work. Please ensure you have included the full name of the authority that approved the field site access and, if no permits were required, a brief statement explaining why.

3. Please include a complete copy of PLOS’s questionnaire on inclusivity in global research in your revised manuscript. Our policy for research in this area aims to improve transparency in the reporting of research performed outside of the researchers’ own country or community. The policy applies to researchers who have travelled to a different country to conduct research, research with Indigenous populations or their lands, and research on cultural artefacts. The questionnaire can also be requested at the journal’s discretion for any other submissions, even if these conditions are not met.  Please find more information on the policy and a link to download a blank copy of the questionnaire here: https://journals.plos.org/plosone/s/best-practices-in-research-reporting. Please upload a completed version of your questionnaire as Supporting Information when you resubmit your manuscript.

4. We note that the grant information you provided in the ‘Funding Information’ and ‘Financial Disclosure’ sections do not match.

When you resubmit, please ensure that you provide the correct grant numbers for the awards you received for your study in the ‘Funding Information’ section.

5. Thank you for stating in your Funding Statement:

“Data collection was funded by the Fisheries and Oceans Canada (DFO) Competitive Science Research Fund under project FS-22-04-02 (EVB, Principal Investigator). DR was supported by a Discovery Grant and the Canada Research Chairs Program. PS was supported by the Chaire de recherche sur les espèces aquatiques exploitées (CREAE-UQAC). RJ was supported by a partial PhD grant from L’Institut Agro. The funders had no role in study design, data collection and analysis, decision to publish, or preparation of the manuscript.”

Please provide an amended statement that declares *all* the funding or sources of support (whether external or internal to your organization) received during this study, as detailed online in our guide for authors at http://journals.plos.org/plosone/s/submit-now.  Please also include the statement “There was no additional external funding received for this study.” in your updated Funding Statement.

Please include your amended Funding Statement within your cover letter. We will change the online submission form on your behalf.

6. Please note that funding information should not appear in any section or other areas of your manuscript. We will only publish funding information present in the Funding Statement section of the online submission form. Please remove any funding-related text from the manuscript.

7. When completing the data availability statement of the submission form, you indicated that you will make your data available on acceptance. We strongly recommend all authors decide on a data sharing plan before acceptance, as the process can be lengthy and hold up publication timelines. Please note that, though access restrictions are acceptable now, your entire data will need to be made freely accessible if your manuscript is accepted for publication. This policy applies to all data except where public deposition would breach compliance with the protocol approved by your research ethics board. If you are unable to adhere to our open data policy, please kindly revise your statement to explain your reasoning and we will seek the editor's input on an exemption. Please be assured that, once you have provided your new statement, the assessment of your exemption will not hold up the peer review process.

8. Your ethics statement should only appear in the Methods section of your manuscript. If your ethics statement is written in any section besides the Methods, please move it to the Methods section and delete it from any other section. Please ensure that your ethics statement is included in your manuscript, as the ethics statement entered into the online submission form will not be published alongside your manuscript.

9. We note that you have referenced (DFO, unpublished data) which has currently not yet been accepted for publication. Please remove this from your References and amend this to state in the body of your manuscript: (ie “Bewick et al. [Unpublished]”) as detailed online in our guide for authors

http://journals.plos.org/plosone/s/submission-guidelines#loc-reference-style

10. We note that Figures 1 and 4 ; Supporting Figures 2, 3, and 4 in your submission contain map images which may be copyrighted. All PLOS content is published under the Creative Commons Attribution License (CC BY 4.0), which means that the manuscript, images, and Supporting Information files will be freely available online, and any third party is permitted to access, download, copy, distribute, and use these materials in any way, even commercially, with proper attribution. For these reasons, we cannot publish previously copyrighted maps or satellite images created using proprietary data, such as Google software (Google Maps, Street View, and Earth). For more information, see our copyright guidelines: http://journals.plos.org/plosone/s/licenses-and-copyright.

We require you to either (1) present written permission from the copyright holder to publish these figures specifically under the CC BY 4.0 license, or (2) remove the figures from your submission:

1. You may seek permission from the original copyright holder of Figure 1 and 4; Supporting Figures 2, 3, and 4 to publish the content specifically under the CC BY 4.0 license.

We recommend that you contact the original copyright holder with the Content Permission Form (http://journals.plos.org/plosone/s/file?id=7c09/content-permission-form.pdf) and the following text:

“I request permission for the open-access journal PLOS ONE to publish XXX under the Creative Commons Attribution License (CCAL) CC BY 4.0 (http://creativecommons.org/licenses/by/4.0/). Please be aware that this license allows unrestricted use and distribution, even commercially, by third parties. Please reply and provide explicit written permission to publish XXX under a CC BY license and complete the attached form.”

Please upload the completed Content Permission Form or other proof of granted permissions as an "Other" file with your submission.

In the figure caption of the copyrighted figure, please include the following text: “Reprinted from [ref] under a CC BY license, with permission from [name of publisher], original copyright [original copyright year].”

2. If you are unable to obtain permission from the original copyright holder to publish these figures under the CC BY 4.0 license or if the copyright holder’s requirements are incompatible with the CC BY 4.0 license, please either i) remove the figure or ii) supply a replacement figure that complies with the CC BY 4.0 license. Please check copyright information on all replacement figures and update the figure caption with source information. If applicable, please specify in the figure caption text when a figure is similar but not identical to the original image and is therefore for illustrative purposes only.

The following resources for replacing copyrighted map figures may be helpful:

USGS National Map Viewer (public domain): http://viewer.nationalmap.gov/viewer/

The Gateway to Astronaut Photography of Earth (public domain): http://eol.jsc.nasa.gov/sseop/clickmap/

Maps at the CIA (public domain): https://www.cia.gov/library/publications/the-world-factbook/index.html and https://www.cia.gov/library/publications/cia-maps-publications/index.html

NASA Earth Observatory (public domain): http://earthobservatory.nasa.gov/

Landsat: http://landsat.visibleearth.nasa.gov/

USGS EROS (Earth Resources Observatory and Science (EROS) Center) (public domain): http://eros.usgs.gov/#

Natural Earth (public domain): http://www.naturalearthdata.com/

10. We note that Figure 2 in your submission contain copyrighted images. All PLOS content is published under the Creative Commons Attribution License (CC BY 4.0), which means that the manuscript, images, and Supporting Information files will be freely available online, and any third party is permitted to access, download, copy, distribute, and use these materials in any way, even commercially, with proper attribution. For more information, see our copyright guidelines: http://journals.plos.org/plosone/s/licenses-and-copyright.

We require you to either (1) present written permission from the copyright holder to publish these figures specifically under the CC BY 4.0 license, or (2) remove the figures from your submission:

1. You may seek permission from the original copyright holder of Figure 2 to publish the content specifically under the CC BY 4.0 license.

We recommend that you contact the original copyright holder with the Content Permission Form (http://journals.plos.org/plosone/s/file?id=7c09/content-permission-form.pdf) and the following text:

“I request permission for the open-access journal PLOS ONE to publish XXX under the Creative Commons Attribution License (CCAL) CC BY 4.0 (http://creativecommons.org/licenses/by/4.0/). Please be aware that this license allows unrestricted use and distribution, even commercially, by third parties. Please reply and provide explicit written permission to publish XXX under a CC BY license and complete the attached form.”

Please upload the completed Content Permission Form or other proof of granted permissions as an "Other" file with your submission.

In the figure caption of the copyrighted figure, please include the following text: “Reprinted from [ref] under a CC BY license, with permission from [name of publisher], original copyright [original copyright year].”

2. If you are unable to obtain permission from the original copyright holder to publish these figures under the CC BY 4.0 license or if the copyright holder’s requirements are incompatible with the CC BY 4.0 license, please either i) remove the figure or ii) supply a replacement figure that complies with the CC BY 4.0 license. Please check copyright information on all replacement figures and update the figure caption with source information. If applicable, please specify in the figure caption text when a figure is similar but not identical to the original image and is therefore for illustrative purposes only.

11. Please include captions for your Supporting Information files at the end of your manuscript, and update any in-text citations to match accordingly. Please see our Supporting Information guidelines for more information: http://journals.plos.org/plosone/s/supporting-information.

12. If the reviewer comments include a recommendation to cite specific previously published works, please review and evaluate these publications to determine whether they are relevant and should be cited. There is no requirement to cite these works unless the editor has indicated otherwise.

13. Please review your reference list to ensure that it is complete and correct. If you have cited papers that have been retracted, please include the rationale for doing so in the manuscript text, or remove these references and replace them with relevant current references. Any changes to the reference list should be mentioned in the rebuttal letter that accompanies your revised manuscript. If you need to cite a retracted article, indicate the article’s retracted status in the References list and also include a citation and full reference for the retraction notice.

[Note: HTML markup is below. Please do not edit.]

Reviewers' comments:

Reviewer's Responses to Questions

**Comments to the Author**

1. Is the manuscript technically sound, and do the data support the conclusions?

Reviewer #1: Yes

Reviewer #2: Yes

**********

2. Has the statistical analysis been performed appropriately and rigorously?

Reviewer #1: Yes

Reviewer #2: Yes

**********

3. Have the authors made all data underlying the findings in their manuscript fully available?

The PLOS Data policy

Reviewer #1: Yes

Reviewer #2: Yes

**********

4. Is the manuscript presented in an intelligible fashion and written in standard English?

Reviewer #1: Yes

Reviewer #2: Yes

**********

Reviewer #1: The authors undertake an ambitious application of otolith chemistry in understanding natal homing by a key forage fish, capelin, within the large Gulf of St. Lawrence system. They navigate adroitly the issue of system/population size through tactics that link adult to early life chemical signatures. A key assumption that across annual cohorts, the early life signature is stable and regrettably could not be tested within the frame of the study. Still the authors sufficiently communicate this limitation throughout the paper. The paper is well presented and analysis appropriate to the study objectives. I found very little to improve in the paper. A couple suggestions:

1. It only became clear to me late in the discussion why more robust and flexible machine learning approaches were not used in classifying individuals. Rather a more standard finite stock mixture approach was used – QDA. I think it would be helpful to present this justification earlier in Methods.

2. The section on integrating connectivity (partial migration) into management (l 436) merits including but the question of how to assess whether this diversity really matters is left untreated. Here there are ways forward through simulation analyses. I list several related citations below that bear on the portfolio effect:

Kerr, L.A., Cadrin, S.X., Secor, D.H., 2010. The role of spatial dynamics in the stability, resilience, and productivity of an estuarine fish population. Ecological Applications, 20(2): 497-507.

Kerr, L.A., Cadrin, S.X., Secor, D.H., Taylor, N.G., 2017. Modeling the implications of stock mixing and life history uncertainty of Atlantic bluefin tuna. Canadian Journal of Fisheries and Aquatic Sciences, 74(11): 1990-2004.

Secor, D.H., Kerr, L.A., Cadrin, S.X., 2009. Connectivity effects on productivity, stability, and persistence in a herring metapopulation model. ICES Journal of Marine Science, 66(8): 1726-1732.

Reviewer #2: Please see the attached review for my detailed comments, as the full text exceeds the character limit.

Overall, I found the manuscript well written and the study valuable for understanding the spatial structure and spawning ecology of capelin in the Gulf of St. Lawrence. The large otolith dataset provides important insights into the relationship between inferred early-life origin and capture regions during and after spawning. My main comments concern the terminology and scope of the movement-related interpretations, as well as some aspects that could be clarified.

**********

what does this mean?). If published, this will include your full peer review and any attached files.

If you choose “no”, your identity will remain anonymous but your review may still be made public.

**Do you want your identity to be public for this peer review?**  For information about this choice, including consent withdrawal, please see our Privacy Policy

Reviewer #1: No

Reviewer #2: No

**********

[NOTE: If reviewer comments were submitted as an attachment file, they will be attached to this email and accessible via the submission site. Please log into your account, locate the manuscript record, and check for the action link "View Attachments". If this link does not appear, there are no attachment files.]

To ensure your figures meet our technical requirements, please review our figure guidelines: https://journals.plos.org/plosone/s/figures

You may also use PLOS’s free figure tool, NAAS, to help you prepare publication quality figures: https://journals.plos.org/plosone/s/figures#loc-tools-for-figure-preparation.

NAAS will assess whether your figures meet our technical requirements by comparing each figure against our figure specifications.

Attachment Submitted filename: Review_JacetalPLOSOne.docx

# Author comment: Author response to Decision Letter 1

**DOI:** 10.1371/journal.pone.0352922.r002

7 Aug 2026

Response to reviewers

We would like to sincerely thank the reviewers for their careful evaluation and constructive feedback,

which have greatly contributed to improving the quality and clarity of our manuscript. We have

carefully considered all comments and made the corresponding revisions throughout the text. In our

responses below, line numbers refer to the revised, clean version of the manuscript (i.e., without

tracked changes).

Reviewer 1

General comments

The authors undertake an ambitious application of otolith chemistry in understanding natal homing by

a key forage fish, capelin, within the large Gulf of St. Lawrence system. They navigate adroitly the issue

of system/population size through tactics that link adult to early life chemical signatures. A key

assumption that across annual cohorts, the early life signature is stable and regrettably could not be

tested within the frame of the study. Still the authors sufficiently communicate this limitation

throughout the paper. The paper is well presented and analysis appropriate to the study objectives. I

found very little to improve in the paper

Answer: We thank the reviewer for their positive assessment of our study and agree that the temporal

stability of the early-life (core) chemical signature is a key premise of our approach.

Specific comments

It only became clear to me late in the discussion why more robust and flexible machine learning

approaches were not used in classifying individuals. Rather a more standard finite stock mixture

approach was used – QDA. I think it would be helpful to present this justification earlier in Methods.

Answer: We agree that the rationale for using QDA reads more naturally where the method is first

introduced rather than emerging later in the Discussion. We have added this justification to the

Methods (lines 197-200).

The section on integrating connectivity (partial migration) into management (l 436) merits including

but the question of how to assess whether this diversity really matters is left untreated. Here there are

ways forward through simulation analyses. I list several related citations below that bear on the

portfolio effect:

Kerr, L.A., Cadrin, S.X., Secor, D.H., 2010. The role of spatial dynamics in the stability, resilience, and

productivity of an estuarine fish population. Ecological Applications, 20(2): 497-507.

Kerr, L.A., Cadrin, S.X., Secor, D.H., Taylor, N.G., 2017. Modeling the implications of stock mixing and

life history uncertainty of Atlantic bluefin tuna. Canadian Journal of Fisheries and Aquatic Sciences,

74(11): 1990-2004.

Secor, D.H., Kerr, L.A., Cadrin, S.X., 2009. Connectivity effects on productivity, stability, and persistence

in a herring metapopulation model. ICES Journal of Marine Science, 66(8): 1726-1732.

Answer: We thank the reviewer for these relevant references and agree that a quantitative test of

whether behavioural/spatial diversity confers a demographic benefit would strengthen this discussion.

Simulation-based population models such as those of Secor et al. (2009), Kerr et al. (2010), and Kerr et

al. (2017) are powerful tools for testing portfolio-effect hypotheses, but in each of these applications

the modelled components are demographically distinct, with vital rates (productivity, natural

mortality, recruitment, growth, maturation) that can be estimated or reasonably assumed to differ.

For capelin in the GSL, we do not currently have evidence for this level of discreteness: genetic

structuring is weak (Cayuela et al., 2020; Kenchington et al., 2015), and behavioural mixing/switching

among putative components appears more extensive than in the systems these models were built for.

Parameterising a simulation under these conditions would require assuming differential productivity

among components largely a priori, which we felt would not yield a robust test of the portfolio-effect

hypothesis at this stage. We have revised the manuscript (lines 410-428) to cite these approaches while

clarifying that establishing demographic differentiation among components is a necessary first step

before such simulation-based methods become applicable to this stock.

Reviewer 2

General comments

I do not agree with classifying individuals as “migratory” or “resident” within the scope of this work.

The analysis compares inferred early-life regional origin with capture region during or after spawning

but does not describe movement throughout the individuals’ lives. Individuals whose inferred early

life and capture regions match may still have migrated before returning to spawn. Therefore, terms

related to “dispersal” or “patterns consistent with regional spawning-site fidelity,” which are also used

in the manuscript, may be more appropriate.

Answer: We thank the reviewer for this important point to which we agree. Our two-point comparison

(inferred early-life region vs. capture region) cannot describe movement across an individual’s full life

history, and a matching region does not rule out movement away from, or back to, that region. We

have removed the “resident”/“migrant” classification throughout the manuscript and replaced it with

language describing only what was measured: whether early-life and capture regions matched or

differed. Where relevant to interpretation, we now use “dispersal” and “patterns consistent with

regional spawning-site fidelity,” as suggested, rather than implying a fixed behavioural status.

In my view, the main findings of this work concern patterns consistent with regional spawning-site

fidelity and the contribution of individuals with early-life signatures assigned to the SBI region (lines

306–314 and 323–325). The opening of the Discussion could focus more clearly on these findings, as

the claims in lines 301–303 appear broader than the scope of the analysis.

Answer: We agree that the original opening framed the study’s scope more broadly (as a first large

scale assessment of movement patterns) than the analysis supports, and buried the two central

findings: (i) patterns consistent with regional spawning-site fidelity and (ii) the distinct local

contribution in the SBI. We have restructured the opening of the Discussion (lines 279-292) to

foreground these two findings directly.

Given the importance of spawning-site fidelity to the study’s findings, I felt that this topic could be

developed more fully in the Introduction. This could include further discussion of the importance of

understanding the species’ spawning ecology, as well as the advantages and limitations of using otolith

chemistry to investigate early-life regional origin and spawning-site fidelity.

Answer: We have added text situating spawning-site fidelity as a behavioural process that can persist

independently of gene flow and that carries potential fitness benefits in the introduction (lines 54-58).

We have also added a brief statement on the advantages of otolith chemistry for investigating early

life origin in a species in lines 65-74.

The term “edge-to-core approach,” used for instance in lines 23, 90 and 209, may lead readers to

assume that the complete life history of each individual was reconstructed along the entire otolith

transect. Please revise this terminology throughout the manuscript and reduce the emphasis placed

on the edge-to-core approach to represent the scope of the analysis accurately.

Answer: We agree that repeated use of "edge-to-core approach" as a named method risked suggesting

reconstruction of an individual's complete life history across the full otolith transect, rather than the

classification of a single core signature against an edge-trained reference model. We have revised the

terminology throughout the manuscript. The method is now introduced in the Methods section, with

an explicit description of what it does; subsequent mentions use the plainer term "core-classification

approach" rather than repeating "edge-to-core" as a standalone label.

Specific Comments

I

found the Methods section somewhat lengthy and presenting some of the information more

concisely could improve readability. For example, the description of the study regions could be moved

to the Introduction and presented alongside the regional otolith-chemistry patterns identified by Jac

et al. (2026).

Answer: We have shortened the Methods section overall.

It would also be helpful to include a descriptive table summarizing the number of samples by region

and year, including spawning phase (spawning or post-spawning), sex, and the mean, minimum and

maximum fish length and age. A table similar to the information presented in S1 Fig would allow

readers to understand the sampling design more easily.

Answer: We have added a descriptive table (Table S1) summarising sample sizes by region, year, and

spawning phase, along with sex composition and the distributions of fork length and age.

The Methods state that this study represents the second attempt to estimate the age of capelin in the

GSL (lines 186–188), but the resulting age estimates are not clearly reported or discussed. Please

present these results or clarify the relevance of this statement.

Answer: As the resulting age estimates were not the focus of this study and were not discussed further,

we have removed this sentence for clarity.

I think it is important to emphasize more clearly that samples were collected from four areas but

assigned to only three classification regions because the chemical signature of the North could not be

distinguished from those of the other regions. Although this information is present in the manuscript,

a clear statement in the Methods, and consistent clarification elsewhere, would help readers follow

the analysis and interpretation.

Answer: We have added an explicit statement at the start of the classification section (lines 192-200)

clarifying that although samples were collected from four areas, the North could not be reliably

distinguished from the other regions based on its chemical signature (Jac et al. 2026, cited in the paper)

and was therefore not retained as a separate classification region, and we have carried this clarification

through consistently elsewhere in the manuscript.

In the interannual comparison (S4 Fig; lines 287–288), the predominant inferred early-life region of

fish captured in the South changes among years, which should be reported in the Results.

Answer: We have revised the relevant sentence (lines 262-264) to explicitly report this exception, i.e.,

that the predominant inferred early-life region of fish captured in the South differed from the general

pattern in 2023.

Although the text explains why sex and maturity were excluded from the final model, I suggest adding

their model-selection results to Table 4 so that readers can assess their contribution directly.

Answer: The model-selection results for sex and maturity have been added to Table 4.

Please include the temperature range in line 394.

Answer: The temperature range has been added.

In the supplementary maps, such as S3 Fig, please clarify that the points are coloured according to

capture site.

Answer: We have clarified this point in the relevant figure captions, which now read: “Points, coloured

by capture region, represent capture locations and are proportional to the number of fish captured.”

Finally, the authors could consider an exploratory clustering analysis of early-life signatures to examine

whether fish of undetermined origin form distinct groups or an intermediate group, potentially

associated with the North.

Answer: While a full clustering analysis is beyond the scope of the present study, we agree that this is

a promising direction for future work. We have added text in the “Uncertainty and limitations” section

of the Discussion (lines 487-491).

Attachment Submitted filename: Response to reviewers.pdf

# Peer review: Decision Letter 1

**DOI:** 10.1371/journal.pone.0352922.r003

**Authors:** Claudio D'Iglio

23 Aug 2026

Regional fidelity vs dispersal of capelin (*Mallotus villosus* ) in the Gulf of St. Lawrence, inferred from otolith chemistry

PONE-D-26-30224R1

Dear Dr. Jac,

We’re pleased to inform you that your manuscript has been judged scientifically suitable for publication and will be formally accepted for publication once it meets all outstanding technical requirements.

Within one week, you’ll receive an e-mail detailing the required amendments. When these have been addressed, you’ll receive a formal acceptance letter and your manuscript will be scheduled for publication.

An invoice will be generated when your article is formally accepted. Please note, if your institution has a publishing partnership with PLOS and your article meets the relevant criteria, all or part of your publication costs will be covered. Please make sure your user information is up-to-date by logging into Editorial Manager at Editorial Manager® and clicking the ‘Update My Information' link at the top of the page. For questions related to billing, please contact billing support.

If your institution or institutions have a press office, please notify them about your upcoming paper to help maximize its impact. If they’ll be preparing press materials, please inform our press team as soon as possible -- no later than 48 hours after receiving the formal acceptance. Your manuscript will remain under strict press embargo until 2 pm Eastern Time on the date of publication. For more information, please contact onepress@plos.org.

Kind regards,

Claudio D'Iglio, Ph.D.

Academic Editor

PLOS One

Additional Editor Comments (optional):

Reviewers' comments:

Reviewer's Responses to Questions

**Comments to the Author**

Reviewer #2: All comments have been addressed

**********

2. Is the manuscript technically sound, and do the data support the conclusions?

Reviewer #2: Yes

**********

3. Has the statistical analysis been performed appropriately and rigorously?

Reviewer #2: Yes

**********

4. Have the authors made all data underlying the findings in their manuscript fully available?

The PLOS Data policy

Reviewer #2: Yes

**********

5. Is the manuscript presented in an intelligible fashion and written in standard English?

Reviewer #2: Yes

**********

Reviewer #2: The authors have made a considerable effort to address the reviewers’ comments, and the manuscript has improved substantially.

In the Methodology section, I recommend retaining the statement that the elemental analyses were conducted at LabMaTer (Université du Québec à Chicoutimi, Canada), as well as the information on the certified reference materials used.

**********

what does this mean?). If published, this will include your full peer review and any attached files.

If you choose “no”, your identity will remain anonymous but your review may still be made public.

**Do you want your identity to be public for this peer review?**  For information about this choice, including consent withdrawal, please see our Privacy Policy

Reviewer #2: No

**********

# Editor report: Acceptance letter

**DOI:** 10.1371/journal.pone.0352922.r004

**Authors:** Claudio D'Iglio

PONE-D-26-30224R1

PLOS One

Dear Dr. Jac,

I'm pleased to inform you that your manuscript has been deemed suitable for publication in PLOS One. Congratulations! Your manuscript is now being handed over to our production team.

At this stage, our production department will prepare your paper for publication. This includes ensuring the following:

* All references, tables, and figures are properly cited

* All relevant supporting information is included in the manuscript submission,

* There are no issues that prevent the paper from being properly typeset

You will receive further instructions from the production team, including instructions on how to review your proof when it is ready. Please keep in mind that we are working through a large volume of accepted articles, so please give us a few days to review your paper and let you know the next and final steps.

Lastly, if your institution or institutions have a press office, please let them know about your upcoming paper now to help maximize its impact. If they'll be preparing press materials, please inform our press team within the next 48 hours. Your manuscript will remain under strict press embargo until 2 pm Eastern Time on the date of publication. For more information, please contact onepress@plos.org.

You will receive an invoice from PLOS for your publication fee after your manuscript has reached the completed accept phase. If you receive an email requesting payment before acceptance or for any other service, this may be a phishing scheme. Learn how to identify phishing emails and protect your accounts at https://explore.plos.org/phishing.

If we can help with anything else, please email us at customercare@plos.org.

Thank you for submitting your work to PLOS One and supporting open access.

Kind regards,

PLOS One Editorial Office Staff

on behalf of

Dr. Claudio D'Iglio

Academic Editor

PLOS One
